# An integrative pan-cancer analysis of the molecular characteristics of dietary restriction in tumour microenvironment

**DOI:** 10.1016/j.ebiom.2024.105078

**Published:** 2024-03-19

**Authors:** Xiaoyi Song, Jiaxing Wei, Yang Li, Wen Zhu, Zhiyuan Cai, Kunwei Li, Jingyue Wei, Jieyu Lu, Wanping Pan, Man Li

**Affiliations:** a Guangdong Provincial Engineering Research Center of Molecular Imaging, The Fifth Affiliated Hospital, Sun Yat-sen University, Zhuhai, 519000, China; b Guangdong-Hong Kong-Macao University Joint Laboratory of Interventional Medicine, The Fifth Affiliated Hospital, Sun Yat-sen University, Zhuhai, 519000, China; c Biobank, The Fifth Affiliated Hospital, Sun Yat-sen University, Zhuhai, 519000, China; d Department of Radiology, The Fifth Affiliated Hospital, Sun Yat-sen University, Zhuhai, 519000, China; e Department of Information Technology and Data Center, The Fifth Affiliated Hospital, Sun Yat-sen University, Zhuhai, 519000, China

**Keywords:** Caloric restriction, Dietary restriction, Pan-cancer, Single-cell transcriptome, Immunotherapy

## Abstract

**Background:**

Dietary restriction (DR), a general term for dieting, has been demonstrated as an effective intervention in reducing the occurrence of cancers. Molecular activities associated with DR are crucial in mediating its anti-cancer effects, yet a comprehensive exploration of the landscape of these activities at the pan-cancer level is still lacking.

**Methods:**

We proposed a computational approach for quantifying DR-related molecular activities and delineating the landscape of these activities across 33 cancer types and 30 normal tissues within 27,320 samples. We thoroughly examined the associations between DR-related molecular activities and various factors, including the tumour microenvironment, immunological phenotypes, genomic features, and clinical prognosis. Meanwhile, we identified two DR genes that show potential as prognostic predictors in hepatocellular carcinoma and verified them by immunohistochemical assays in 90 patients.

**Findings:**

We found that DR-related molecular activities showed a close association with tumour immunity and hold potential for predicting immunotherapy responses in various cancers. Importantly, a higher level of DR-related molecular activities is associated with improved overall survival and cancer-specific survival. FZD1 and G6PD are two DR genes that serve as biomarkers for predicting the prognosis of patients with hepatocellular carcinoma.

**Interpretation:**

This study presents a robust link between DR-related molecular activities and tumour immunity across multiple cancer types. Our research could open the path for further investigation of DR-related molecular processes in cancer treatment.

**Funding:**

10.13039/501100001809National Natural Science Foundation of China (Grant No. 82000628) and the Guangdong-Hong Kong-Macao University Joint Laboratory of Interventional Medicine Foundation of Guangdong Province (Grant No. 2023LSYS001).

**Research in context**

**Evidence before this study**

Dietary restriction has demonstrated remarkable efficacy in cancer prevention and unneglectable potential as an adjuvant to chemotherapy and immunotherapy. We searched PubMed with the terms “Caloric restriction and Pan-cancer,” “Dietary restriction and Pan-cancer,” or “Diet and Pan-cancer” for articles published up to November 10, 2023, with no language restrictions. We found no literature that was similar to our work. Although previous studies had reported the mechanisms underlying the protective effects of dietary restriction in a few cancer types, molecular characteristics of dietary restriction at the pan-cancer level remain unclear. This study provides a comprehensive evaluation of dietary restriction-related molecular activities at the pan-cancer level.

**Added value of this study**

In this study, using a multi-omics analysis that included 33 cancer types, we established and validated a quantitative metric to evaluate the levels of dietary restriction-related molecular activities. At the pan-cancer level, we uncovered the associations between dietary restriction and tumour microenvironment, immune phenotypes, genomic features, and clinical prognosis. Furthermore, intratumoral heterogeneity in dietary restriction-related molecular activities was revealed by analysis of 939,718 single-cell profiles, and these activities were linked to cell–cell paracrine interactions between immune and tumour cells. We proposed a framework for enhancing the understanding of dietary restriction-related modulations in the tumour microenvironment. This framework could stimulate further investigations into dietary restriction-related molecular mechanisms and advance the development of dietary restriction-related personalized therapeutic strategies in clinical oncology.

**Implications of all the available evidence**

Dietary restriction has demonstrated significant efficacy as a non-pharmacological intervention in reducing the occurrence and advancement of both spontaneous and induced cancers. Given the common variation of the molecular biological heterogeneity across different cancer types, it becomes particularly intriguing to investigate dietary restriction's respective roles in diverse types of cancer. Such research endeavours can contribute to the refinement and customization of dietary restriction-related therapy for specific tumours. Furthermore, studying the levels of these molecular activities in patients with cancer who have not undergone dietary restriction intervention can provide insights into how dietary restriction arrives at its anti-cancer effectiveness. Comprehensive characterization of dietary restriction at the pan-cancer level offers valuable insights into the context-dependent regulation of dietary restriction in the tumour microenvironment, shedding light on the dietary restriction-related biomarker discoveries and therapeutic targets.

## Introduction

Dietary restriction (DR), a general term for dieting including caloric restriction, refers to the restriction of nutrient intake while still providing sufficient essential amino acids, vitamins, and nutrients, thus avoiding malnutrition.^1^ DR has proven to be the most effective non-pharmacological intervention for cancer prevention in both rodents and monkeys, and, in humans, it promotes anticarcinogenic adaptations by reducing the production of growth factors, inflammatory cytokines, anabolic hormones, oxidative stress, and free-radical-induced DNA damage.^2^ Additionally, DR has garnered traction as a potential adjuvant to chemotherapy and immunotherapy, suggesting promising translational applications.^3^^,^^4^

However, the mechanisms underlying the anti-cancer benefits of DR are not fully understood in humanity.^5^ Notably, most of the evidence regarding the potentially beneficial anti-cancer effects of DR for cancer management has been collected in preclinical animal models,^6^^,^^7^ in which inconclusive or conflicting conclusions have also been discussed.^8^^,^^9^ On the other hand, despite a wealth of literature on the impact of DR, individual responses to DR can vary.^10^ Previous studies have identified that the mechanisms underlying the protective effects of DR are mainly associated with a reduction in oxidative stress, glucose levels, insulin resistance, insulin-like growth factor 1 (IGF-1), and growth hormone (GH) levels.^11^ These molecular activities play a significant role in mediating the anti-cancer effects of DR and are closely associated with DNA repair and autophagy.^12^^,^^13^ On the contrary, DR can inhibit pathways that are positively related to tumour growth and reduce the carcinogenic and metastatic potential of cancer stem cells, which are generally responsible for tumour formation and relapse.^14^ The combined effect of these positive and negative modulations determines the anti-cancer effects of DR.

Given the common variation of these molecular and biological activities across different cancer types, it becomes particularly intriguing to investigate their respective roles in diverse types of cancer. Such research endeavours can contribute to the refinement and customization of DR-related therapy for specific tumours. Furthermore, studying the levels of these molecular activities in patients with cancer who have not undergone DR intervention can provide insights into how DR arrives at its anti-cancer effectiveness.

In this study, we developed and validated a quantitative metric to assess the levels of DR-related molecular activities through a comprehensive multi-omics analysis encompassing 33 cancer types and 30 normal tissues. We identified the specific associations of DR with genomic variations and immune molecular characteristics at pan-cancer level. Additionally, our investigation of 939,718 single-cell profiles reveals that DR-related molecular activities exhibit intratumoral heterogeneity and are associated with cell–cell paracrine communications between tumour cells and immune cells. Moreover, the levels of DR-related molecular activities show significant associations with tumour immunomodulation and have potential for predicting immunotherapy responses in various cancer types. Importantly, our findings indicate that a higher level of DR-related molecular activities is associated with improved overall survival (OS) and cancer-specific survival (CSS). In summary, our integrated analyses offer valuable insights into the context-dependent regulation of DR-related molecular activities in the tumour microenvironment, shedding light on the DR-related biomarker discoveries and therapeutic targets.

## Methods

### Data and resources

Somatic mutation data (mutation annotation format), RNA-seq data (STAR-Counts), and associated clinical data for 33 cancer types (9938 samples) were downloaded from the Cancer Genome Atlas (TCGA) database using the R package 'TCGAbiolinks' (version 2.25.2).^15^ Proteomic data in the TCGA and the gene expression matrix of 30 tissue types (17,382 samples) and corresponding information in the Genotype-Tissue Expression (GTEx) database were collected from UCSC Xena (http://xena.ucsc.edu/public/). The gene expression matrix of 1348 cell-lines of 31 cancer types in Cancer Cell Line Encyclopedia (CCLE) database was obtained from DepMap Data (http://depmap.org/portal/download/all/). Expression profiles of immunotherapy cohorts were retrieved through accession numbers (SKCM_LUAD_LUSC_GSE93157, SKCM_PRJEB23709, GBM_PRJNA482620, SKCM_GSE115821, SKCM_Nathanson2017, STAD_PRJEB25780).^16^, ^17^, ^18^, ^19^, ^20^ Expression profiles and corresponding survival information from two LIHC validation cohorts were obtained from the project ‘LIRI-JP’ of the International Cancer Genome Consortium (ICGC) and GSE14520.^21^ Expression profiles and corresponding grouping information from the DR-related clinical trial and the obesity-related cohort were obtained from GSE63117 and GSE69039,^22^^,^^23^ respectively.

Single-cell transcriptome data were collected from the Gene Expression Omnibus (GEO) database (https://www.ncbi.nlm.nih.gov/geo/) and Tumor Immune Single-cell Hub (TISCH) database (http://tisch.comp-genomics.org/home/).^24^ Single-cell RNA-seq data of different cancers contain BRCA (GSE161529, 332,168 cells), CHOL (GSE138709, 33,990 cells), CRC (GSE166555, 66,050 cells), ESCA (GSE160269, 208,658 cells), KIRC (GSE159115, 27,669 cells), NSCLC (GSE117570, 11,453 cells), OV (EMTAB8107, 32,386 cells), PAAD (CRA001160, 57,443 cells), PRAD (GSE176031, 18,807 cells), and STAD (GSE134520, 41,554 cells).^25^, ^26^, ^27^, ^28^, ^29^, ^30^, ^31^, ^32^, ^33^, ^34^ Single-cell RNA-seq data of immunotherapy cohorts contains BCC (GSE123813, 52,884 cells), BLCA (GSE145281, 14,474 cells), SCC (GSE123813, 25,891 cells), and SKCM (GSE120575, 16,291 cells).^35^, ^36^, ^37^

Spatial transcriptome (ST) data were obtained from Datadryad (https://doi.org/10.5061/dryad.h70rxwdmj). A total of 16 pathological sections and 51,850 spots from 16 patients with GBM were collected.^38^

The tumour purity score, tumour ploidy score, neoantigen score, homologous recombination deficiency (HRD), and homologous recombination deficiency-loss of heterozygosity (LOH) of TCGA samples were obtained from the UCSC Xena database and a previous study.^39^ Microsatellite instability (MSI) of TCGA samples was obtained from the previous study.^40^

### Dietary restriction score development

We obtained a total of 276 DR-related genes (Table S1) from The Dietary Restriction Gene Database (GenDR).^41^ The genes were divided into two gene sets: 220 positive DR-related genes and 56 negative DR-related genes. We utilized the ‘gsva’ function of the ‘GSVA’ packages,^42^ with the parameters set as follows: (1) method = “ssgsea”; (2) kcdf = “Poisson”; (3) min. sz = 10, to calculate the normalized enrichment scores (NES) of two gene sets in individual samples, respectively. The DR score was defined as the differences between the positive DR-related score (NES1) and the negative DR-related score (NES2). We defined the DR score in individual samples as follows: DR score = NES1–NES2. The significance levels of DR scores were estimated from the permutation test by randomly selecting several genes that were the same as the gene number used to construct the DR signature and replicated 1000 times in one sample. Tumor samples were divided into high-DR and low-DR groups by the median of DR scores. For single-cell RNA-seq datasets and the spatial dataset, we utilized the same Gene Set Variation Analysis (GSVA) method as described above to calculate the DR score for every single cell or spot.

To validate the robustness of the DR score, we performed non-ranked gene set enrichment analysis of the DR genes in the gene ontology gene sets and pathways gene sets by function ‘TCGAanalyze_EAcomplete’ of the R package ‘TCGAbiolinks’. The pathways gene sets can be obtained by function ‘TCGAbiolinks:::listEA_pathways’. Additionally, we calculated the GSVA scores of C6 oncogenic signature gene sets of the Molecular Signatures Database (MSigDB) for all TCGA samples and CCLE cell lines and then performed Spearman correlations between the DR scores and these GSVA scores.

### Single-cell transcriptome analysis

The selection criteria for scRNA-seq datasets were as follows: (1) solid tumours; (2) contain both tumour samples and corresponding normal samples; (3) sample size ≥5 and total cells >10,000; (4) contain complete conventional components in the tumour microenvironment, including the malignant/epithelial cells, stroma cells, and immune cells. For each obtained dataset, a uniform analysis pipeline—MAESTRO^43^ was adopted to perform quality control, batch effect removal, clustering, and cell-type annotation. The criteria of cell quality control were as follows: (1) nFeatures >200; (2) the mitochondrial gene ratio <20%. We conducted data integration by removing the confounding factors in each dataset by R package ‘harmony’^44^ and processed data normalization, cell clustering, and visualization. Cells were annotated based on reported marker genes accordingly. The DEGs between the high-DR and low-DR groups in cancer cells and immune cells were identified separately by function ‘FindMarkers’ with default parameters. Then, the significant DEGs (adjusted *P*-value <0.05 and average fold-change >1) were collected to perform the GSEA analysis. The cell–cell paracrine communications analysis was performed using the R package ‘CellChat’^45^ with the count matrix as the input. Significant interactions paired were selected with *P*-value <0.05 (Wilcoxon rank-sum test).

### Spatial transcriptome analysis

For each image in the obtained dataset, a pipeline—SpaCET^46^ was adopted to perform the downstream analysis, including quality control, cell type deconvolution and annotation, region division, and cell–cell interactions inference. Spots without UMI counts were filtered out, while the eligible spots were deconvoluted and annotated based on SpaCET built-in reference matrix. Then, the tumour–stroma interface of each image was identified by function ‘SpaCET.identify.interface’, and the spots were then grouped into ‘Interface’, ‘Stroma’, and ‘Tumour’. As for the analysis of cell–cell paracrine communications in ST data, we constructed the ligand-receptor (L-R) interaction network by function ‘SpaCET.CCI.LRNetworkScore’ and used the network score to represent the overall intensities of L-R interactions at each spot.

### Investigating dietary restriction-related immune characteristics at pan-cancer level

Firstly, we utilized single sample gene set enrichment analysis (ssGSEA) algorithm deconvoluted the compositions of 16 immune cells in tumours and quantified the activities of 13 immunological functioning pathways by reference gene sets obtained from a previous study.^47^ Then, ESTIMATE^48^ algorithm was utilized to evaluate the levels of total infiltration of immune cells with the gene expression matrix (TPM matrix) as input and default settings. Moreover, the absolute abundances of 22 immune cell types in TCGA samples were inferred by CIBERSORT^49^ algorithm, with gene expression matrix (TPM matrix) and LM22 signature as the reference.

Patient clusters were identified in TCGA-LIHC and ICGC-LIRI-JP cohorts using the unsupervised consensus clustering algorithm in R package ‘ConsensusClusterPlus’^50^ with default settings. The optimal k was determined by the relative change of the areas under the CDF curves. All the immune-related pathways in the KEGG used to perform the GSEA analysis on the expression matrix of the patient clusters were obtained from C2 canonical pathways in MSigDB.

The IC_50_ of the chemotherapy and targeted agents was calculated using the function ‘pRRopheticPredict’ in ‘pRRophetic’ R package.^51^ We used TPM matrix as the input with the built-in dataset ‘cgp2016’ as the reference.

### Genomic variation analysis

For single nucleotide variants (SNVs), the total numbers of mutation events in TCGA samples were collected using function ‘getSampleSummary’ in R package ‘maftools’.^52^ As the key assumptions of linear regression were satisfied, the associations between the DR scores and mutation load were analysed by multiple linear regression models that had been adjusted for age at diagnosis, sex, and race. Benjamini-Hochberg adjusted *P*-values were calculated to control the false positive. The specific mutation events of TCGA samples were further analysed and visualized in R package ‘maftools’ with default parameter settings.

Copy number variations (CNVs) were analysed using GISTIC 2.0^53^ to identify arm- and focal-level alterations in 25 TCGA cancer types with at least 100 samples. Significant broad events were considered alterations occurring in >70% of one arm with q values < 0.25. Chromosome-level events were considered events in which both arms had the same log2 ratio of copy number. We defined CNV scores based on the previous method.^54^ For arm-level events, the log2 ratios of copy number were transferred into arm-level scores as follows: 2 if log2 ratio ≥1; 1 if log2 ratio ≥0.25 and < 1; 0 if log2 ratio ≥ −0.25 and < 0.25; −1 if log2 ratio ≥ −1 and < −0.25; −2 if log2 ratio < −1. The arm score was the sum of all arm-level scores in one sample. The focal scores and chromosome scores were calculated using a similar procedure. The overall CNV score of each sample was defined as the sum of arm, focal, and chromosome scores. As the key assumptions of linear regression were satisfied, the associations between the DR scores and CNV scores were analysed using similar multiple linear regression models as with SNP. Benjamini-Hochberg adjusted *P*-values were calculated to control the false positive. Under the condition that all logistic regression assumptions were satisfied, the associations between the DR scores and CNV gains or losses were analysed using logistic regression models adjusting for age at diagnosis, sex, and race. Arm-level gains and losses were defined based on the log2 ratio of copy-number >0.25 and < −0.25, respectively.

### Construction of LIHC-DR predictor

A total of 815 patients in 3 LIHC cohorts were utilized to develop and validate the LIHC-DR predictor. Prognosis-related genes in the DR signature of patients with LIHC were chosen by the least absolute shrinkage and selection operator (LASSO), random forest and boruta (RFB), and extreme gradient boosting regressor (XGBoost). LASSO algorithm was conducted by function ‘cv.glmnet’ in R package ‘glmnet’^55^ using a 5-fold cross-validation and we selected the tuning parameters by ‘lambda.min’. RFB was implemented by function ‘TentativeRoughFix’ in R package ‘Boruta’. XGBoost was performed by function ‘xgb.train’ in R package ‘xgboost’. For all the three algorithms, the OS-related Cox regression was fitted. The common genes (FZD1 and G6PD) that were derived by all the three algorithms were used to construct a multiple Cox regression model.

### Survival analysis

The survival disparities between the low-DR and high-DR groups were evaluated through Kaplan–Meier survival curves. The log-rank test was employed to identify significant differences, with a *P*-value <0.05 deemed statistically significant. Survival analyses were performed using the R packages ‘survival’ and ‘survminer’. For all the survival analyses, the origin and start times were the same. The time of diagnosis of disease was set as the starting point and the time of death (or tumor-specific death) was set as the endpoint of the survival analysis. If there was no death reported for a patient before the data cutoff analysis, the OS (or CSS) was censored at the last contact date at which the patient was known to be alive. All the events and censored data for each dataset were reported (Table S2). Under the condition that all Cox regression assumptions were satisfied, Cox proportional hazards regression models of OS and CSS at the pan-cancer level were constructed by setting DR scores, sex, cancer types, and age at diagnosis as variables. HR [95% confidence interval (CI)] and *P*-values were visualized by R packages ‘epiDisplay’.

### Immunohistochemistry stain

We obtained two tissue chips (HLivH180Su30) comprising 90 LIHC tumour specimens along with corresponding follow-up information from Shanghai Outdo Biotech Co, Ltd (Shanghai, China). Paraffin-embedded tissue sections were completely dewaxed in xylene and subsequently rehydrated using a series of ethanol gradients (100% and 95%). Antigen retrieval was performed by heating the sections for 15 min at 95–98 °C in sodium citrate buffer. The sections were then incubated overnight at 4 °C with primary antibodies. Primary antibodies, including anti-FZD1 (rabbit polyclonal LS-A4150, 1:200, LS-A3484-50, LSBio, AB_591,407), anti-G6PD (rabbit polyclonal, 1:500, ab993, Abcam, RRID: AB_296714) were used. Comprehensive assessment of secondary antibodies was biotinylated labelled anti-rabbit antibodies (UltraSensitiveTM SP Rabbit Kit-9706/9707/9708, MXB Biotechnologies). We used QuPath program^56^ to evaluate the staining intensities and expression percentages of these proteins on slides, while the H-score for each slide was generated to represent the protein expression level in slides. The clinical demographic data of the patients in this cohort is detailed in Table S3.

### Statistical analysis

Continuous data were compared using appropriate statistical tests: Student's *t*-test, Wilcoxon rank-sum test, or Kruskal–Wallis test depending on whether the distribution of the data conformed to normal distribution. We employed the QQ plot and Levene's test to examine the normality of the data and equality of variances, respectively. Categorical data were analysed using the Chi-square test, and none of the expected cell counts of less than 5 occurred in the comparative analysis throughout this study. The Spearman correlation coefficient was utilized to assess variable correlations. The log-rank test was employed to determine statistical significance in survival analysis. The diagnostic efficiency of prediction models was quantified by the area under the receiver operating characteristic curve (AUC) and the 95% CI of AUC was calculated using 10,000 bootstrap replicates. A *P*-value <0.05 was considered indicative of a significant difference for all statistical tests. R software (version 4.1.0) was utilized for all statistical analyses.

### Role of the funding source

The funders did not play any role in the study design, data collection, management, analysis, interpretation, review, approval of the manuscript, or the decision to submit the manuscript for publication.

## Results

### Quantification of DR-related molecular activities

**Fig. 1:**
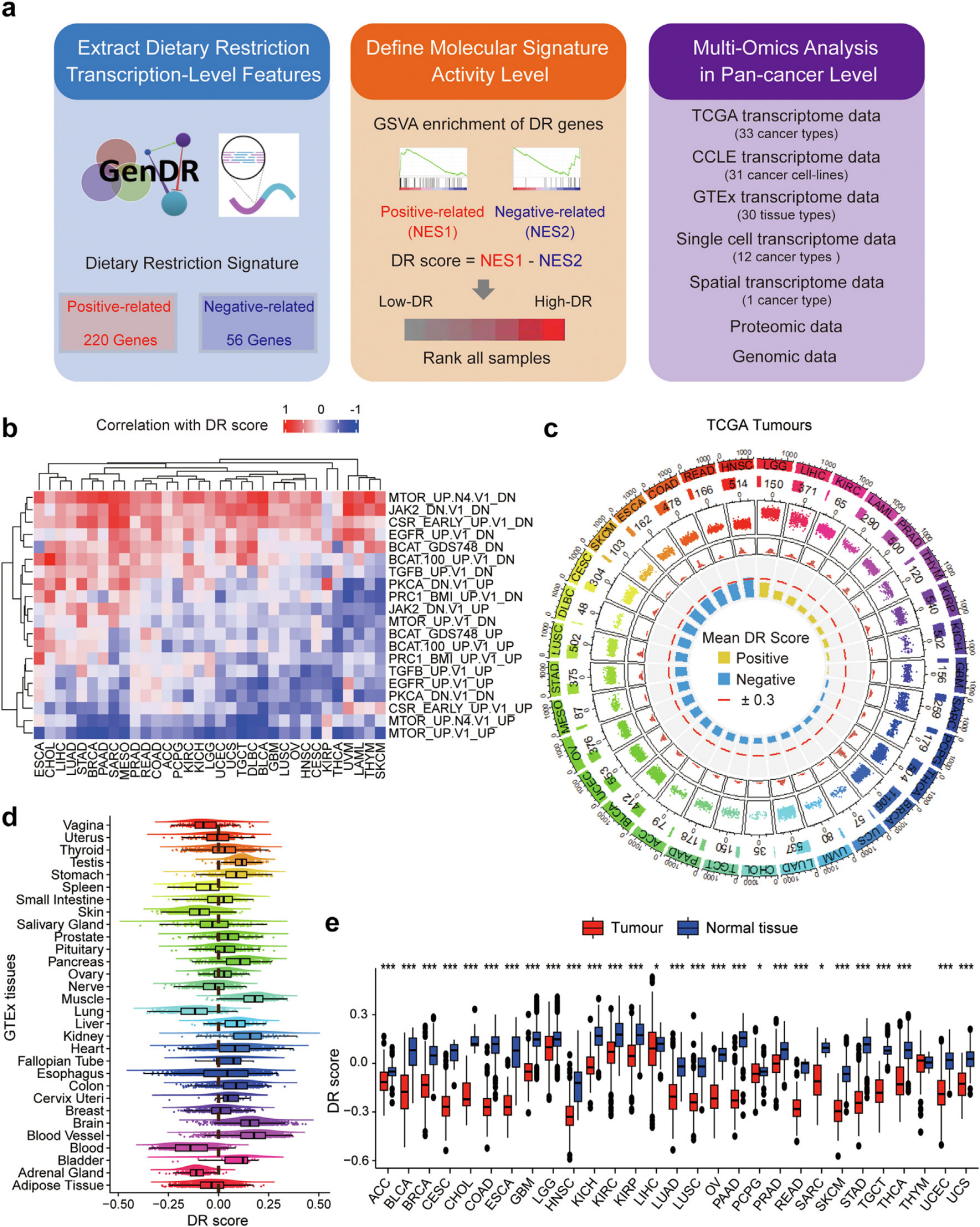
**Comprehensive quantification of DR-related molecular activities in cancers and tissues**. (a) Overall methodology. Workflow for integrative analysis of the DR landscape across cancers using multi-omics data. NES, normalized enrichment score. (b) Heatmaps showing correlations between the DR score and GSVA scores of oncogenic processes across 33 TCGA cancer types. (c) Average DR scores in individual cancer types. Cancer types and average DR scores are shown from the inner circle to the outer circle. (d) Average DR scores across normal tissues in the GTEx dataset. (e) Lower DR scores in primary tumours (red) in comparison to normal solid tissues (blue). In Box-and-whisker plots, an outlier is defined as a data point located outside the whiskers that are outside 1.5 times the interquartile range above the upper quartile and below the lower quartile. The *P*-values (Wilcoxon rank-sum test) are stated: ∗∗∗ indicates *P* < 0.0001; ∗∗ indicates *P* < 0.01; ∗ indicates *P* < 0.05.

To quantify DR-related molecular activities, we obtained a comprehensive set of 276 DR-related genes from GenDR database. The GenDR database comprises two datasets: (1) Genes identified through genetic manipulation experiments that nullify or disrupt the effects of DR. We considered these genes to be positively related to DR. (2) Genes that consistently show significant alterations in response to DR based on a meta-analysis of microarray studies in mammals. For this dataset, we classified genes that were up-regulated by DR implementation as positively related to DR, while those that were down-regulated were deemed negatively related to DR. We hypothesized^57^ that a more active DR-related molecular signature would yield more pronounced differences between the activities of genes positively related to DR and those negatively related to DR. We employed the GSVA algorithm to infer the activities of genes that were positively and negatively linked with DR in each sample, and then we defined a “DR score”, which could reflect the discrepancy between these two sets of activities (Fig. 1a).

We first assessed the reliability of DR score as a representative of DR-related molecular activities. We found that DR genes were mainly enriched in processes involving AMP-activated protein kinase (AMPK), NRF2-mediated oxidative stress response, mammalian target of rapamycin (mTOR), and NF−κβ signalling that have been reported to mediate the anti-cancer effects of DR^58^, ^59^, ^60^, ^61^ (Figure S1a). To further validate the DR score, we obtained 38 paired blood samples (GSE63117) from 19 normal donors from a DR-related clinical trial (NCT00561145), in which the participants were subjected to a two-week weight maintenance diet, followed by three weeks of 30% caloric restriction. As anticipated, the DR scores of the DR-treated group were significantly higher than those of the control group (Figure S1b). Upon GSVA algorithm, we also examine the effect of DR on the main enrichment pathways of DR genes. Consistent with previous reports, DR activates AMPK signalling as well as inhibits NRF2-mediated oxidative stress response, mTOR signalling, and NF−κβ signalling (Figure S1c). Additionally, DR score exhibited negative correlations with the activities of oncogenic signatures both in TCGA and CCLE databases, including JAK2-, EGFR-, and PKCA-regulated genes (Fig. 1b and Figure S1d). These results align with the current consensus that DR is associated with a decreased risk of cancer.^62^

Then, we calculated DR scores in 9938 samples across 33 cancer types in the TCGA database and 1348 cell lines across 31 cancer types in the CCLE database. The distribution of DR-related molecular activities specific to each cancer type is shown in Fig. 1c and Figure S1e. Generally, the majority of cancer types in both the TCGA (24 out of 33) and CCLE (27 out of 31) databases had an average DR score below 0. To compare cancer-specific distribution with DR scores in normal tissues, we investigated DR scores in 17,382 samples across 30 types of tissues from the GTEx database (Fig. 1d). In contrast to tumours, most normal organs (21 out of 30) exhibited an average DR score above 0. Importantly, almost all cancer types in the TCGA showed significantly lower DR scores in tumour tissues compared to their corresponding normal tissues, suggesting that elevated levels of DR-related molecular activities in the tumour microenvironment may counteract tumour growth (Fig. 1e). Interestingly, tissues commonly associated with active glucose metabolisms, such as brain, liver, kidney, and thyroid tumours, exhibited relatively higher DR scores in both the TCGA and GTEx cohorts. The consistent tendency of DR scores in both TCGA and GTEx suggests that tissue type may play a crucial role in determining the extent of DR-related molecular activities.

Considering the similarity between DR-regulated molecular pathways and those associated with obesity,^63^ we proceeded to examine the link between DR and obesity. Our analysis incorporated a cohort (GSE69039) comprising 18 blood samples from normal donors spanning a range of body mass index (BMI) categories: Normal (18.5–23 kg/m^2^), Overweight (25–27.5 kg/m^2^), and Obesity (27.5–30 kg/m^2^). We observed an inverse relationship between BMI and DR scores (Figure S2a). We extended our inquiry to the correlation of DR scores with fundamental demographic variables within the TCGA cohort, where only samples with available data on age, sex, and weight were analysed. The correlations of DR scores with age, sex, and weight were found to be weak (Spearman index <0.4), indicating that DR score may serve as an independent metric for assessing DR, unaffected by these baseline characteristics (Figure S2b). To determine if the DR signature can forecast obesity-associated malignancies, we stratified cancer samples from TCGA into two distinct groups per existing evidence^64^: an obesity-related cohort and an unrelated cohort (Figure S2c). Comparative analysis showed that samples from the unrelated cohort exhibited elevated DR scores compared to those in the obesity-related cohort (*P* < 0.0001, Student's *t*-test, Figure S2d).

### Heterogeneity of DR score and the association with tumour immune microenvironment

**Fig. 2:**
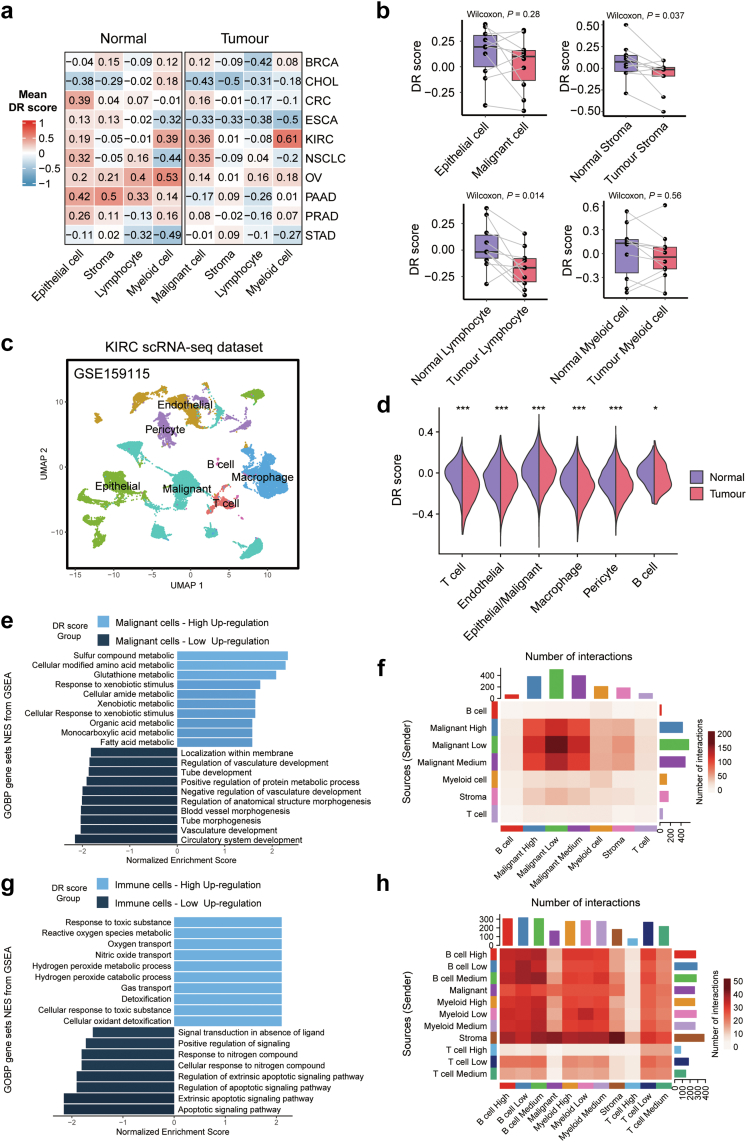
**Heterogeneity of DR-related molecular activities in tumour microenvironments**. (a) Heatmaps showing the average DR scores of different cell types (epithelial cells, stromal cells, lymphocyte cells, and myeloid cells) in 10 single-cell datasets, which are labelled as the cancer type. (b) The comparison of DR score in different major cell lineages between tumours and corresponding normal tissues. In Box-and-whisker plots, an outlier is defined as a data point located outside the whiskers that are outside 1.5 times the interquartile range above the upper quartile and below the lower quartile. (c) Uniform manifold approximation and projection plot shows main cell types in single-cell kidney renal clear cell carcinoma data (GSE159115), coloured by cell type. The *P*-values (Wilcoxon rank-sum test) are stated: ∗∗∗ indicates *P* < 0.0001, ∗∗ indicates *P* < 0.01, and ∗ indicates *P* < 0.05. (d) The comparison of DR score in different cell subtypes between tumours and corresponding normal tissues. (e) Bar plots showing the top 10 enriched GO pathway terms of DEGs in malignant cells between the high-DR (light blue) and low-DR groups (dark blue). (f) Heatmaps showing the number of ligand-receptor interactions in the tumour microenvironments at single-cell level based on the malignant cells categories. (g) Bar plots showing the top 10 enriched GO pathway terms of DEGs in immune cells between the high-DR (light blue) and low-DR groups (dark blue). (h) Heatmaps showing the number of ligand-receptor interactions in the tumour microenvironments at single-cell level based on the immune cells categories.

We employed 10 scRNA-seq datasets with a total of 830,178 cells across 10 types of solid tumours from 173 patients with cancer as well as 65 normal donors to evaluate the heterogeneity of DR scores and the association between DR scores and tumour microenvironment. Consistent with bulk RNA-seq data, cells in normal tissues generally exhibited higher DR scores compared to those in the tumour microenvironment (Fig. 2a). Furthermore, we observed higher DR scores in 4 cell lineages when comparing tumour and normal cells, with statistical significance observed only in stromal cells and lymphocyte cells (Fig. 2b). To explore DR score heterogeneity across different cancer types, we compared the DR scores of distinct cell types in normal tissues and tumours (Fig. 2c and d, and Figure S3). As anticipated, most stromal and immune cells within the tumour microenvironment exhibited lower DR scores compared to their counterparts in normal tissues, suggesting a preference for reduced DR-related molecular activities in the tumour microenvironment. This may also indicate that tumour cells could rob surrounding normal tissue cells of nutrients,^65^ resulting in decreased DR scores in these cells.

To investigate the impact of intratumoral heterogeneity of DR-related molecular activities in tumour microenvironment, we analysed DR scores in 15,000 cells from 8 patients with KIRC (GSE159115). The cancer cells and the immune cells in this dataset were stratified into high-DR, medium-DR, and low-DR (based on the quartile of DR scores, high-DR: >75%; medium-DR: 25%–75%; low-DR: <25%) groups separately. Our findings revealed a strong correlation between elevated levels of DR score and tumour cell metabolism (Fig. 2e). Additionally, the high-DR groups displayed enhanced responses to xenobiotic stimuli (mostly referring to the response to drug), potentially influencing the chemotactic activities of immune cells (Fig. 2e). Furthermore, a lower level of DR score within tumours was associated with increased tumour angiogenesis, suggesting that low-DR tumour cells may contribute significantly to tumour growth (Fig. 2e). Moreover, cell–cell paracrine communication analysis revealed a negative relationship between DR score and the number of cell–cell interactions among tumour cells. Tumor cells in the low-DR group showed the highest number of interactions, indicating their heightened activity within the tumour microenvironment (Fig. 2f). Meanwhile, immune cells in the high-DR group were engaged in the cellular oxidant detoxification process, which is essential for the oxidation-reduction system and has associations with antitumorigenesis.^66^, ^67^, ^68^ The immune cells in the low-DR group exhibited significant upregulation of genes related to the extrinsic apoptosis signalling pathway (Fig. 2g). Furthermore, we found an increased number of interactions between the immune cells in the low-DR group and the tumour cells, suggesting that the immune cells in the low-DR group experienced substantial influence from the tumour cells (Fig. 2h). We further investigated the altered PD-1/PDL1 activity in the immune cells and tumor cells at the transcription level. We found that CD274 was mainly expressed in tumor cells, whereas the expression of PDCD1 in the immune cells in the low-DR group was higher than that of those in the high-DR group (Figure S4). Additionally, the expression of PDCD1 in immune cells gradually increased with the decrease in DR scores.

**Fig. 3:**
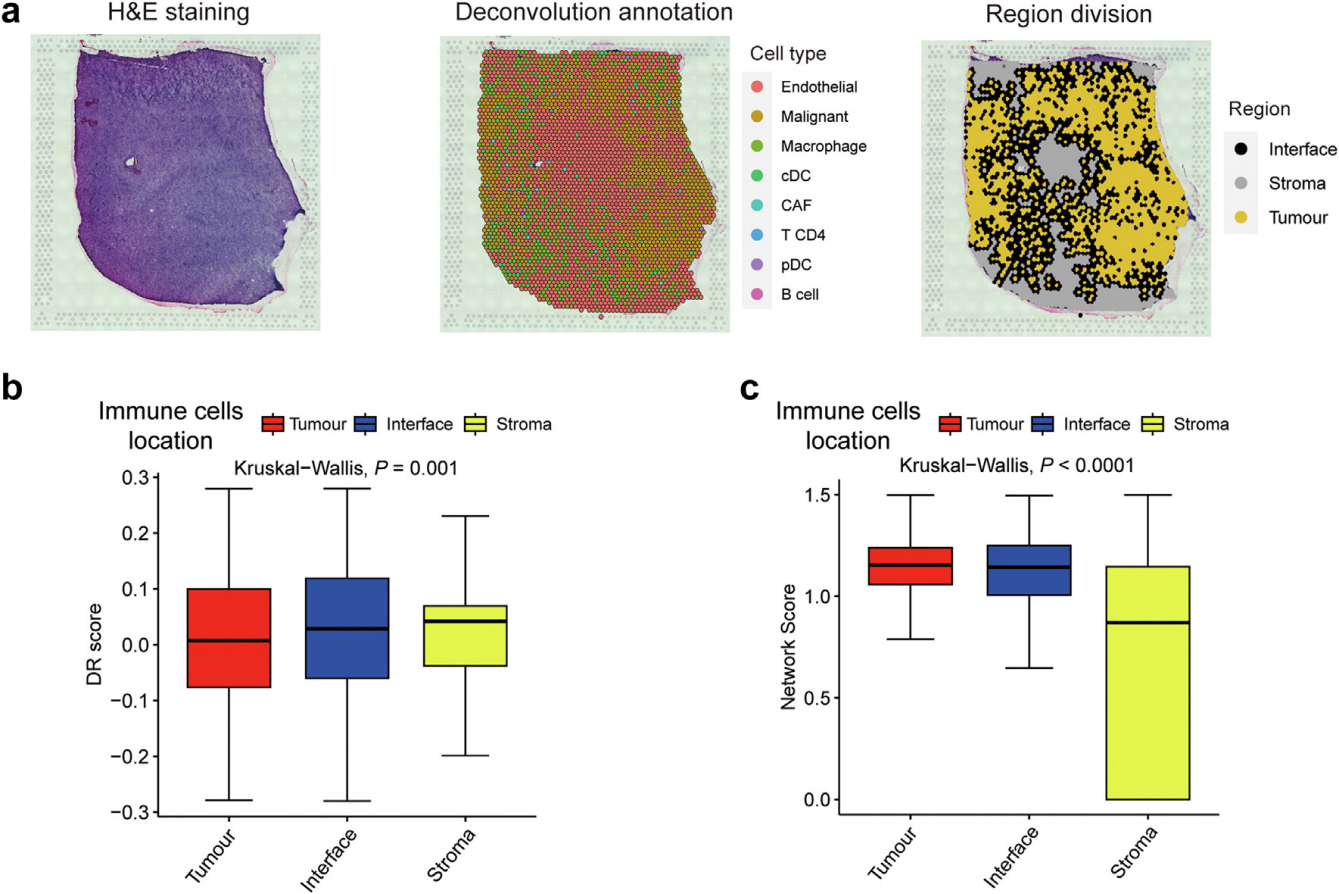
**Spatial transcriptome reveals the associationbetweenDR scores and the tumour immune microenvironment**. (a) Overall methodology. Workflow for integrative analysis of the spatial transcriptome data. (b) The DR scores of the immune cells in different spatial distributions (tumor, interface, stroma). (c) The association between the DR scores of the immune population and the single-cell spatial interactions of immune cells.

We then utilized a ST dataset^38^ with a total of 16 pathological sections and 51,850 spots from 16 patients with GBM to further examine the correlation between DR score and tumour immune microenvironment at the spatial level. Based on the spatial distribution of immune cells, we categorized them into three groups: inside the tumour, on the interface, and in the stroma (Fig. 3a). The immune cells within the tumour exhibited the lowest average DR score, whereas those in the stroma displayed the highest average DR score (Fig. 3b). As the proximity of immune cells to tumour cells increased, a significant decline in DR scores was observed (*P* = 0.001, Kruskal–Wallis test). Furthermore, through cell–cell interactions, we found that immune cells within tumours had higher network scores compared to those in other locations, indicating their active engagement in communication with tumours (Fig. 3c). These results highlighted the ability of DR scores to reflect the active status of immune cells within tumour microenvironment, emphasizing the distinct status of immune cells compared to those in normal tissue.

### Association of DR with cancer type-specific genomic variations

Genomic instability is widely recognized as a characteristic of both cancer and aging.^69^ However, the extent to which genomic alterations may vary with the level of DR-related molecular activities across different types of cancer remains to be elucidated. In this study, we investigated the associations between DR scores and two types of genomic alterations: SNVs and CNVs.

**Fig. 4:**
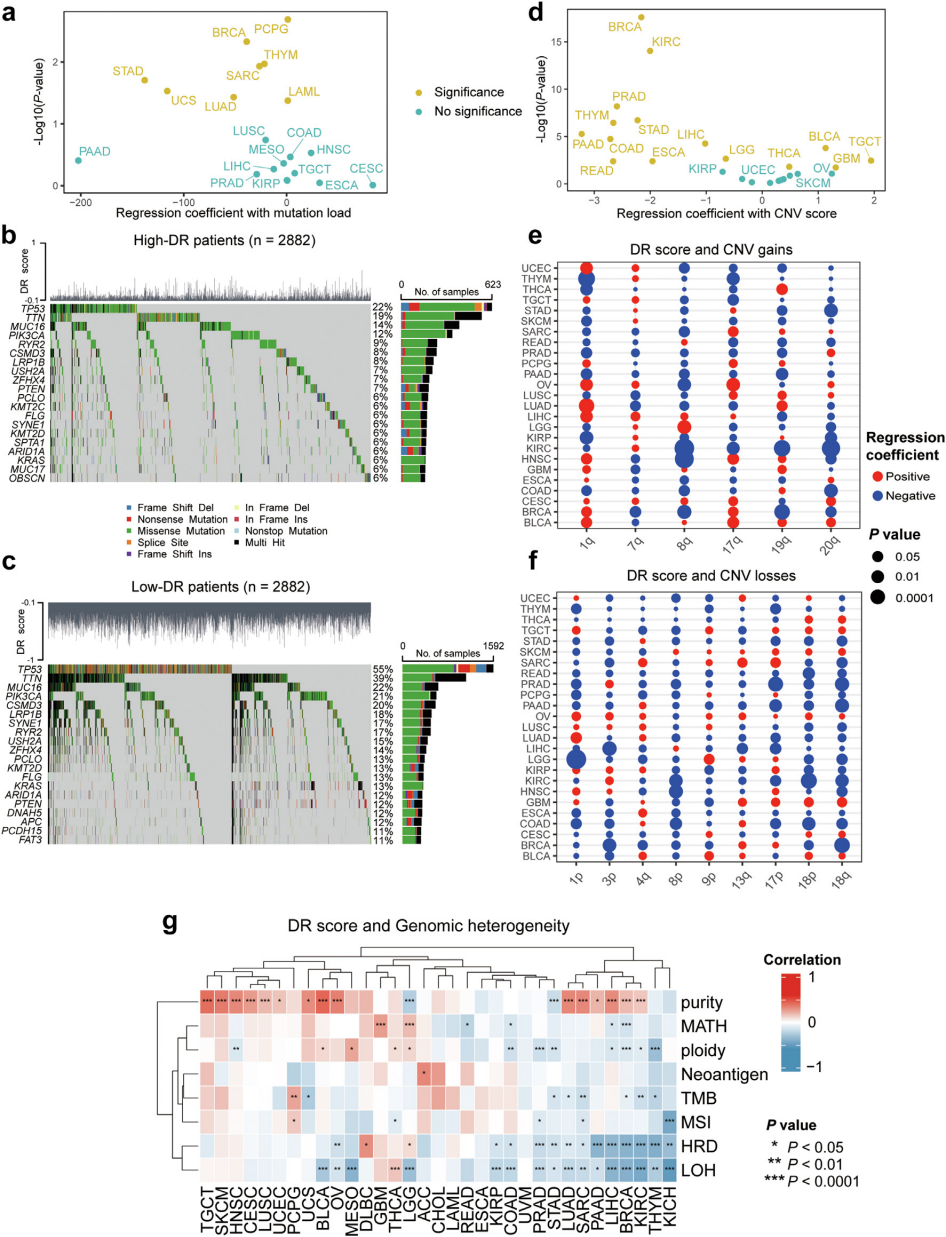
**Associations between DR scores and genomic variations at the pan-cancer level**. (a) Associations between DR scores and mutation loads across TCGA cancer types. Regression coefficients and significance [−log10 (Benjamini-Hochberg-adjusted *P*-values)] are shown on the x-axis and y-axis. Coloured yellow are significant DR-related cancers (Adjusted *P*-values <0.05, F-test) (b and c) Heatmap showing the top mutation events for individual patients with cancer of TCGA cohort in the high-DR (b) and low-DR groups (c), respectively. Bar plots in the top panel represent the DR scores of individual patients. A statistical graph of mutation events for each gene is shown in the left panel. Colours are variant classifications. (d) Associations between DR scores and CNV scores across TCGA cancer types. Regression coefficients and significance are shown on the x-axis and y-axis. Coloured yellow are significant DR-related cancers (Adjusted *P*-values <0.05, F-test) (e and f) Dot plots showing the associations between DR scores and arm-level CNV gains (e) and CNV losses (f). Circle size indicates significance [−log10 (Benjamini–Hochberg-adjusted *P*-values)], and the circle colour denotes coefficients in multiple logistic regression. (g) Heatmap showing the correlation between DR scores and various indexes of genomic heterogeneity. The *P*-values (Spearman rank correlation test) are labelled: ∗∗∗ indicates *P* < 0.0001, ∗∗ indicates *P* < 0.01, and ∗ indicates *P* < 0.05.

For SNVs, we initially examined the relationship between DR scores and the mutation load by a multiple linear regression model that had been adjusted for age, sex, and race. The cancer-specific analyses revealed significantly negative correlations between DR scores and the mutation load in 8 cancer types (Fig. 4a). To gain a deeper understanding, we further analysed the differences in specific mutational events in TCGA patients between the low-DR and high-DR groups (grouped by the median of DR score). Generally, missense mutations were the predominant mutation event in both groups, with the majority being C > T mutations (Figure S5a and b). Compared to high-DR group, the low-DR group had a higher number of alterations per sample (84 in low-DR vs 34 in high-DR). Subsequently, we separately examined the top 20 mutated oncogenic genes in each group (Fig. 4b and c). TP53, TTN, MUC16, and PIK3CA mutations were the top four mutation events observed in both groups, but the mutation frequency of these genes in the low-DR group was nearly twice that of the high-DR group. Our data revealed that the PIK3CA of the tumours in low-DR group may have a higher probability of harmful mutations that make cancer cells less responsive to DR.^70^ Additionally, among the top 20 mutated oncogenic genes in the low-DR group, we identified mutations that closely correlated with tumour progression, such as those in APC and FAT3,^71^^,^^72^ indicating a more malignant characteristic of the tumours in low-DR patients.

In a similar manner to SNVs, we observed a negative correlation between the DR scores and the occurrence of CNV events in almost half of the cancer types (11 out of 25), such as LIHC [coef (95% CI): −1.02 (−1.51, −0.53)], KIRC [coef (95% CI): −2.00 (−2.49, −1.51)], and COAD [coef (95% CI): −2.71 (−3.94, −1.49)] (Fig. 4d). Generally, the majority of arm-level CNV events were negatively correlated with DR scores across cancer types, with arm-level gains showing a higher association with DR scores than arm-level losses (Figure S6a and b). Furthermore, we identified cancer type-specific arm-level CNV events that were relatively concentrated in certain chromosome arms in patients with LIHC that had lower DR scores (Fig. 4e and f). Previous studies reported that the amplification of 19q and 20q, as well as the deletion of 3p, 13q, and 17p, correlated with metastasis and poor prognosis in patients with LIHC.^73^ This suggests that LIHC tumours with low DR scores may exhibit a more aggressive phenotype.

We further investigated the correlation between genomic heterogeneity and DR scores. As shown in Fig. 4g, we found a positive correlation between DR scores and tumour purity across most cancer types, which aligns with our single-cell analysis revealing decreased DR scores in non-tumor cells within the tumour microenvironment. Additionally, despite the heterogeneity, DR scores were negatively correlated with tumour ploidy, tumour mutational burden (TMB), HRD, and LOH in several cancer types, including LIHC, KIRC, and BRCA. These findings suggest that specific cancer types, such as LIHC, with low DR scores are more likely to exhibit genomic instability.

### Association of DR with immune infiltration and prognosis

**Fig. 5:**
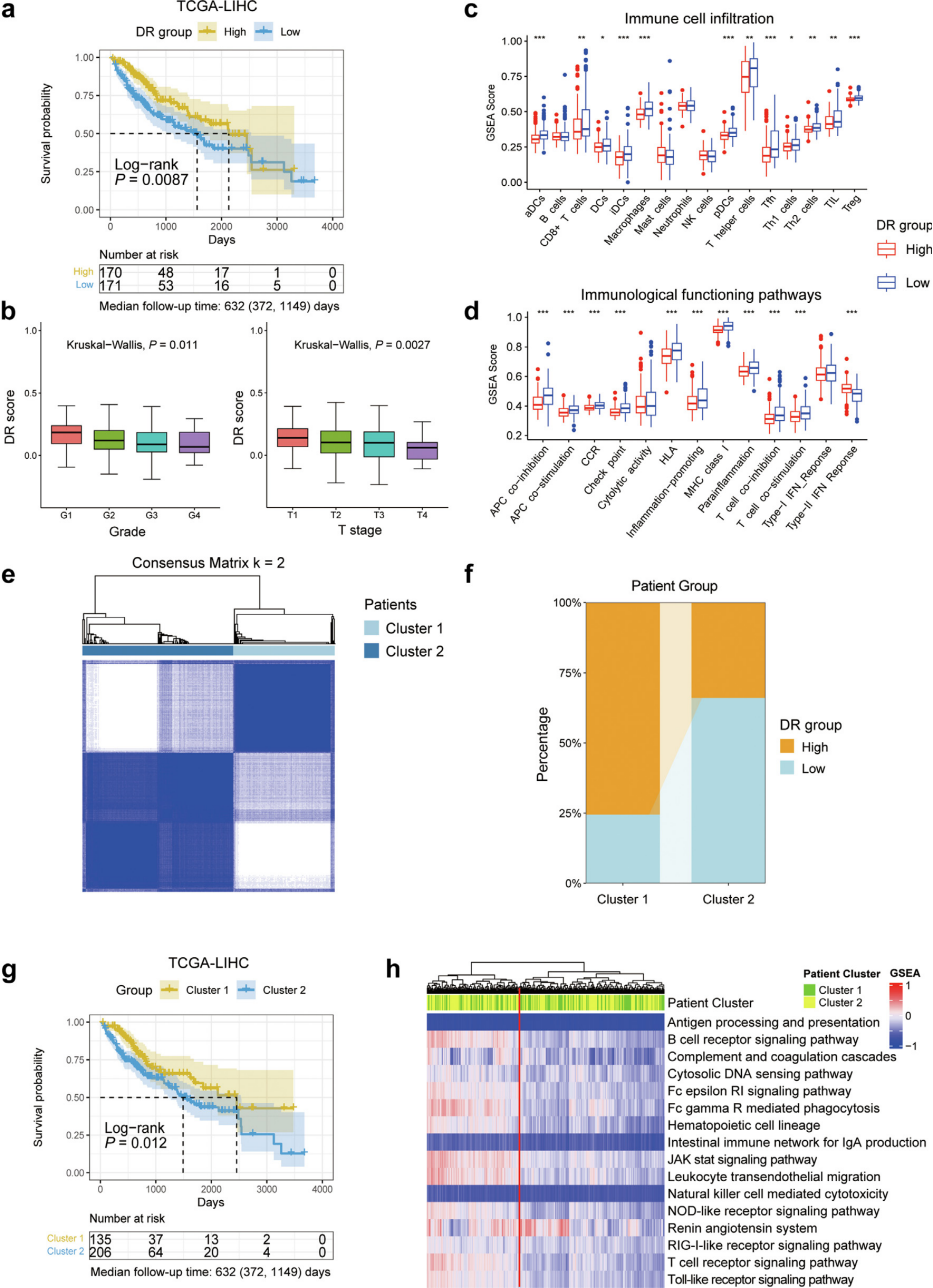
**Identifying the clinical relevance of DR scores**. (a) Kaplan–Meier curves of OS for the high and low groups stratified by the median DR score in TCGA-LIHC. The shaded area refers to the 95% confidence band for survival curves. (b) Box plots showing the DR score across tumour grade (left) and T stages (right) of patients in the TCGA-LIHC (c and d) Deconvolution analysis quantifies the immune infiltration of 16 immune cells in tumours (c) and the activities of 13 immunological functioning pathways (d) within high-DR and low-DR groups, respectively. In Box-and-whisker plots, an outlier is defined as a data point located outside the whiskers that are outside 1.5 times the interquartile range above the upper quartile and below the lower quartile. The *P*-values (Wilcoxon rank-sum test) are stated: ∗∗∗ indicates *P* < 0.0001; ∗∗ indicates *P* < 0.01; ∗ indicates *P* < 0.05. (e) Consensus clustering identified two distinct clusters based on the DR signature in transcriptome data of patients with LIHC (k = 2). (f) Barplot showing the high-DR and low-DR groups ratio of the patients in the two consensus clusters. (g) Kaplan–Meier curves of OS for clusters (Cluster 1 and Cluster 2) of patients with LIHC that were identified from the consensus clustering results. The shaded area refers to the 95% confidence band for survival curves. (h) Agglomerative hierarchical clustering of GSEA scores for all immune-related pathways in the KEGG database, annotated by patient cluster in the colour bar. For each survival analysis, the median (IQR) of the follow-up time is reported.

Within the TCGA-LIHC cohort, patients in the high-DR group had significantly better overall survival than those in the low-DR group (Fig. 5a). Furthermore, a higher level of DR-related molecular activities was found to be associated with better tumour grade and T-staging (Fig. 5b). Given that abundant immune infiltration in tumours is commonly regarded as a prognostic factor,^74^ we further investigated the relationship between the DR scores and immune infiltration. Through ssGSEA algorithm, we deconvoluted the compositions of 16 immune cells in tumours and quantified the activities of 13 immunological functioning pathways. Interestingly, patients with high DR scores exhibited a significantly lower level of immune infiltration in their tumours (Fig. 5c). Conversely, patients with low DR scores exhibited increased activity in immunological functioning pathways (Fig. 5d). Similar results were observed in other cancer types (Figure S7).

To validate our observations, we performed consensus cluster analysis based on the transcriptome expression of 276 DR-related genes. The patients with LIHC were categorized into two groups (k = 2) based on the consensus matrix (Fig. 5e). Cluster 1 predominantly comprised patients with high DR scores (approximately 75%), while about 70% of patients in Cluster 2 had low DR scores (Fig. 5f). Kaplan–Meier analysis revealed a poorer prognosis for Cluster 2 patients compared to Cluster 1 (*P* = 0.012, log-rank test, Fig. 5g). Hierarchical cluster analysis of immune-related pathways in the KEGG pathway revealed a prevalent pattern of immune activation pattern in the majority of Cluster 2 patients (Fig. 5h). Similar outcomes were obtained in the ICGC-LIRI-JP cohort, validating our findings (Figure S8a–d). In summary, our findings indicate a negative correlation between DR-related molecular activities and immune infiltration, while positively correlating with prolonged survival.

### Association of DR with immune checkpoints and immunomodulation in pan-cancer

**Fig. 6:**
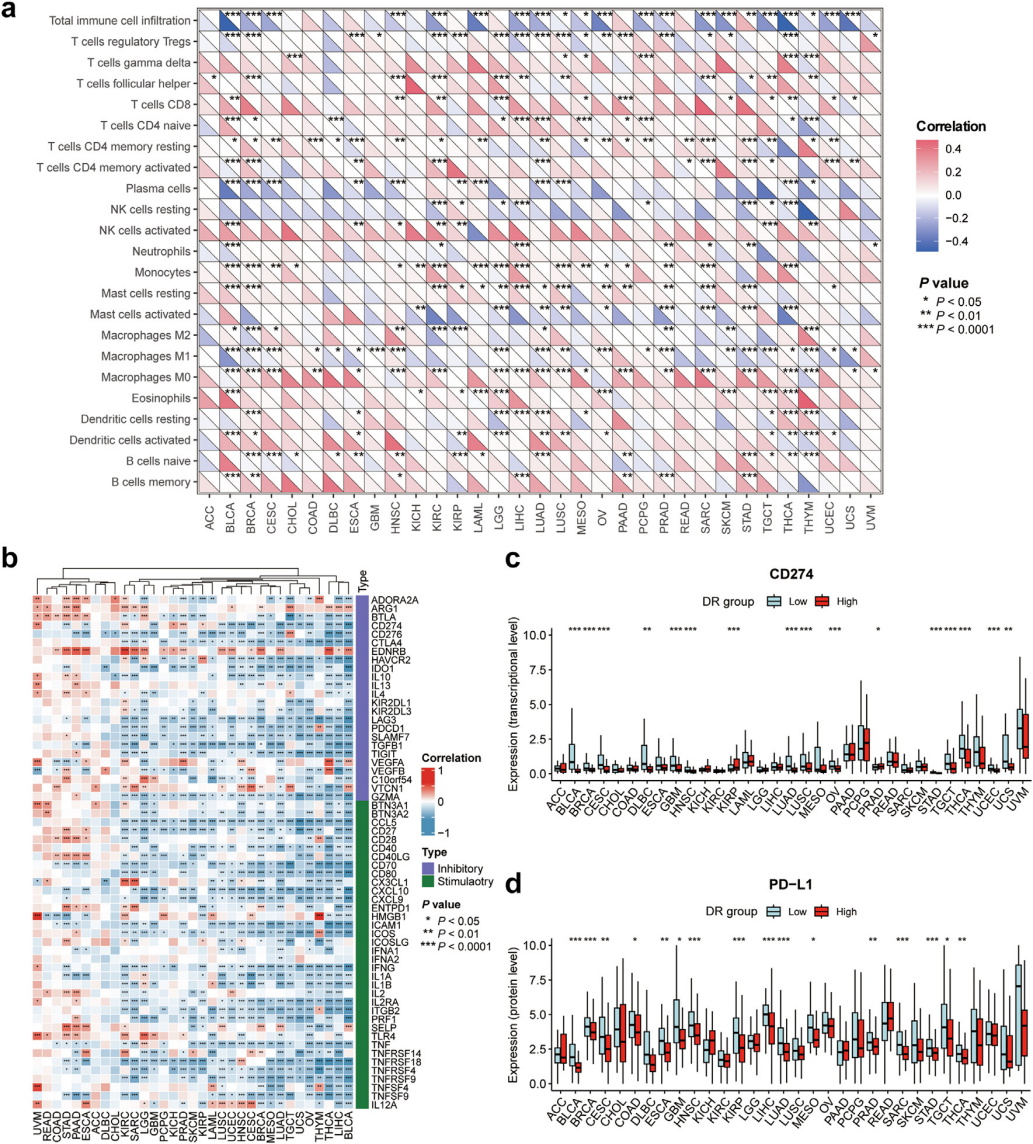
**Cancer type-specific associations of DR scores with tumour immunity**. (a) Spearman correlations between DR scores and the absolute abundance of the total cell immune population and 22 immune cell types estimated by the ESTIMATE algorithm and CIBERSORT algorithm for individual TCGA cancer types. (b) Heatmap showing the Spearman correlations of DR scores with the expression level of well-known inhibitory and stimulatory immune checkpoints. (c and d) Boxplots comparing the differences in PD-L1 expression between the low- and high-DR groups for individual TCGA cancer types at the transcriptional level (c) and protein level (d). The *P*-values (Wilcoxon rank-sum test) are stated: ∗∗∗ indicates *P* < 0.0001, ∗∗ indicates *P* < 0.01, and ∗ indicates *P* < 0.05.

We wondered whether DR-related molecular activities were correlated to the immune checkpoints and immunosuppression in cancers. As expected, the immune infiltration abundances of total immune cells were significantly correlated with DR scores in most cancer types (21 out of 33) and most of them exhibited a negative correlation between the immune infiltration and DR score (Fig. 6a). However, the infiltrations of specific immune cells varied across cancer types, and the correlation between DR score and the immune infiltrations varied correspondingly. For instance, DR score was negatively correlated to the infiltration of immunosuppressive regulatory T cells (Tregs) in LIHC, but not all cancer types. This result suggests the presence of an immunosuppressive microenvironment in tumours of patients with LIHC that had a lower DR score.

To further examine the association between DR and immune characteristics, we analysed the correlation between DR scores and the expression of immune checkpoint genes. As depicted in Fig. 6b, there was a significant negative correlation between DR scores and the expression of immune checkpoint genes in the majority of cancer types. Interestingly, we observed that most cancer types, such as LIHC, LUAD, BLCA, BRCA, and GBM, exhibited enhanced expression of PD-L1 transcripts and proteins in the low DR groups (Fig. 6c and d). Furthermore, we examined the correlation between DR scores and immune cytolytic activity (CYT) scores. The CYT score, which is calculated as the average of granzyme A (GZMA) and perforin (PRF1) transcripts, serves as a biomarker for predicting immune responses by evaluating the infiltration of T-cell cytotoxicity.^75^^,^^76^ As revealed in Figure S9a, the low DR groups consistently displayed higher CYT scores in the majority of cancer types (29 out of 33). Ultimately, our integrated analysis of PD-L1 expression and CYT scores revealed a significant negative correlation with DR scores in eight cancer types, including LUAD, LUSC, BRCA, and BLCA (Figure S9b). These findings suggest that tumours with low DR scores and elevated PD-L1 expression, along with sufficient cytolytic T-cells, may exhibit increased susceptibility to immunotherapy in the corresponding microenvironment.

Our findings suggest that patients exhibiting a low level of DR-related molecular activities might possess a tumour microenvironment characterized by significant immunosuppression, potentially contributing to an unfavourable prognosis. Nevertheless, it is essential to acknowledge that these patients exhibit substantial immune infiltration and robust molecular characteristics associated with anti-tumor immunity. Consequently, the DR score holds promise as a valuable tool for identifying patients with a higher likelihood of favourable response to immunotherapy.

### DR as a potential predictor of immunotherapy response

**Fig. 7:**
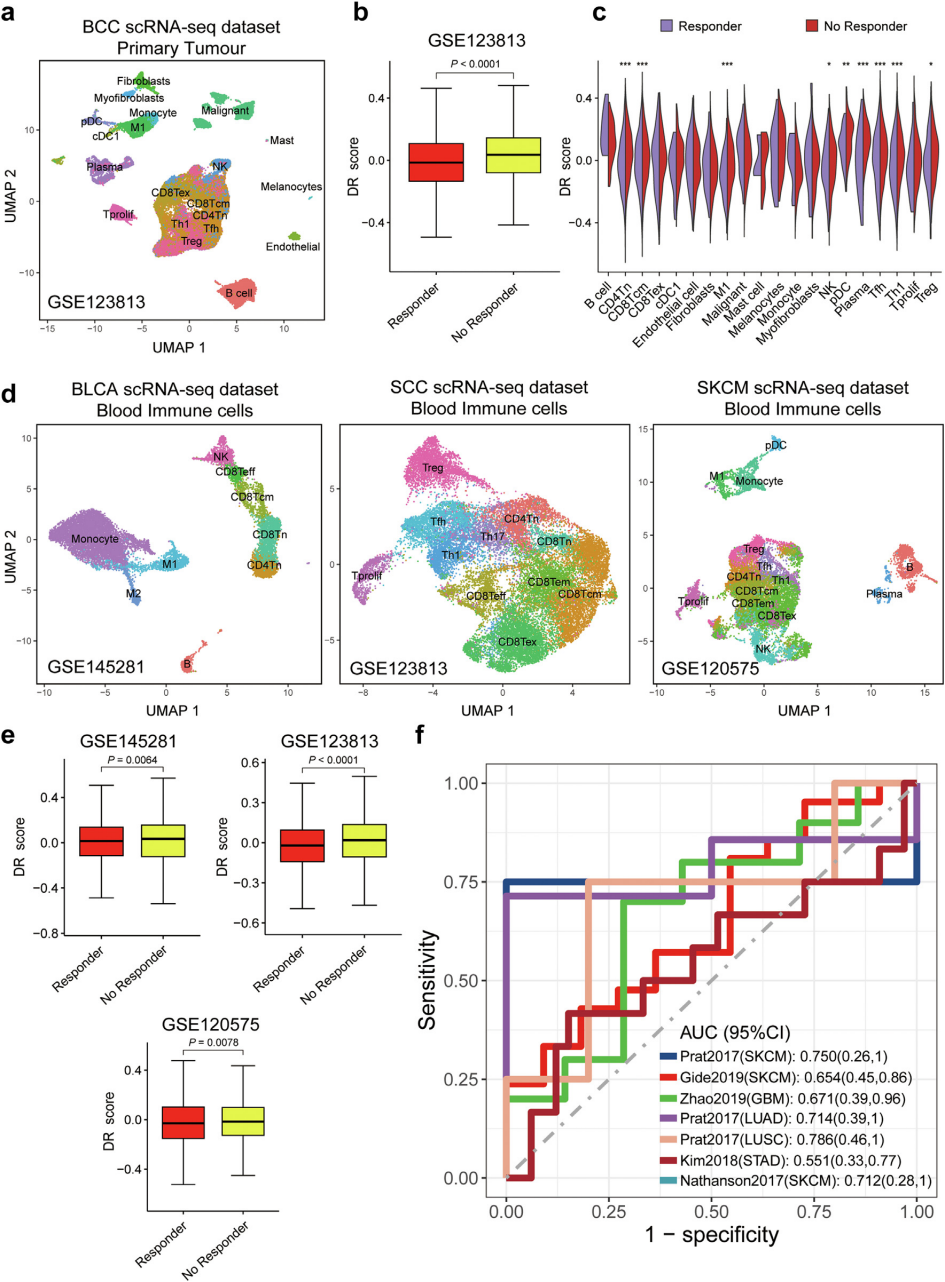
**Associations of DR scores with immunotherapy responses**. (a) Uniform manifold approximation and projection plot showing main cell types in the primary tumour of patients with BCC (GSE123813). (b) Boxplots showing the discrepancy of DR scores in total cells within the GSE123813 datasets of responders and nonresponders before receiving immunotherapy treatment. (c) Violin plots showing the discrepancy of DR scores in cell subtypes within the tumour microenvironment of responders and nonresponders before receiving immunotherapy treatment. (d) Uniform manifold approximation and projection plot showing main cell types in blood samples of patients with BLCA (left, GSE145281), patients with SCC (middle, GSE123813), and patients with SKCM (right, GSE120575) receiving immunotherapy treatment. (e) Boxplots showing the discrepancy of DR scores in immune cells within the three blood-sample datasets of responders and nonresponders before receiving immunotherapy treatment. (f) receiver operating characteristic (ROC) curves of the DR score in distinguishing responders and nonresponders to immunotherapy in seven different cohorts. AUCs were calculated by ROC analysis and are labelled in the bottom right. The 95% confidence intervals of the AUCs are reported in parentheses. The *P*-values (Wilcoxon rank-sum test) are stated: ∗∗∗ indicates *P* < 0.0001, ∗∗ indicates *P* < 0.01, and ∗ indicates *P* < 0.05.

The immune alterations observed in different DR scores led us to believe that DR scores may hold potential in predicting the response to immune checkpoint blockade (ICB) therapy. To gain a deeper insight, we compared the DR scores of primary tumour samples before immunotherapy (21,256 single cells from the GSE123813) between ICB responders and ICB nonresponders in a scRNA-seq dataset of basal cell carcinoma (Fig. 7a). As anticipated, the DR scores of cells from responders were lower compared to those of cells from nonresponders before immunotherapy (Fig. 7b). Further analysis of cell types revealed that the differences in DR scores were mainly observed in immune cells and stroma cells (Fig. 7c). Subsequently, we extended our analysis to compare the DR scores of blood immune cells before immunotherapy (34,804 single cells in GSE145281, GSE123813, and GSE120575) between ICB responders and ICB nonresponders (Fig. 7d). Consistent with the findings observed in solid tumours, we obtained similar results where the DR scores of cells from responders were lower compared to those of cells from nonresponders (Fig. 7e).

Ultimately, we investigated the predictive capability of DR scores in large RNA-seq samples from multiple cohorts consisting of 163 patients across 6 ICB cohorts. DR score achieved a mean AUC of approximately 0.69 for predicting the response of ICB across different cancer types (Fig. 7f). This indicates the potential of DR scores as a predictive tool for identifying ICB responders.

DR may also have the potential to be an adjuvant to chemotherapy and targeted therapy.^13^^,^^77^ With the R package ‘pRRophetic’, we predicted the drug sensitivity value (IC_50_) of 237 available chemotherapies and targeted agents in Cancer Genome Project (CGP2016) for all TCGA samples. Despite the variation, the DR scores exhibited significant correlations with the IC_50_ of most chemotherapy and targeted agents at pan-cancer level (Figure S10). Given that part of the chemotherapy and target therapy effects are mediated by metabolic rewiring (influenced by DR), the effects of DR can be disparate in different drugs. Our result may help enhance the understanding of the role of DR associated with these treatments.

### Construct and validate the dietary restriction-related predictor in hepatocellular carcinoma

Translating the DR signature into the application of clinical practice is the ultimate goal of this study. By adjusting for age at diagnosis, sex, and cancer types in the Cox proportional hazard regression, we identified the DR score was an independent risk predictor for both OS [HR (95% CI): 0.25 (0.16, 0.37)] and CSS [HR (95% CI): 0.18 (0.11, 0.29)] at pan-cancer level (Figure S11a). Moreover, we observed significant statistical differences in OS between low-DR group and high-DR group (divided by the median of DR scores) in seven cancer types (Figure S11b). To further explore the potential clinical value of DR scores in specific cancer types, we next investigated the DR scores in LIHC, a cancer type that usually requires precise caloric intake control during treatment.^78^

**Fig. 8:**
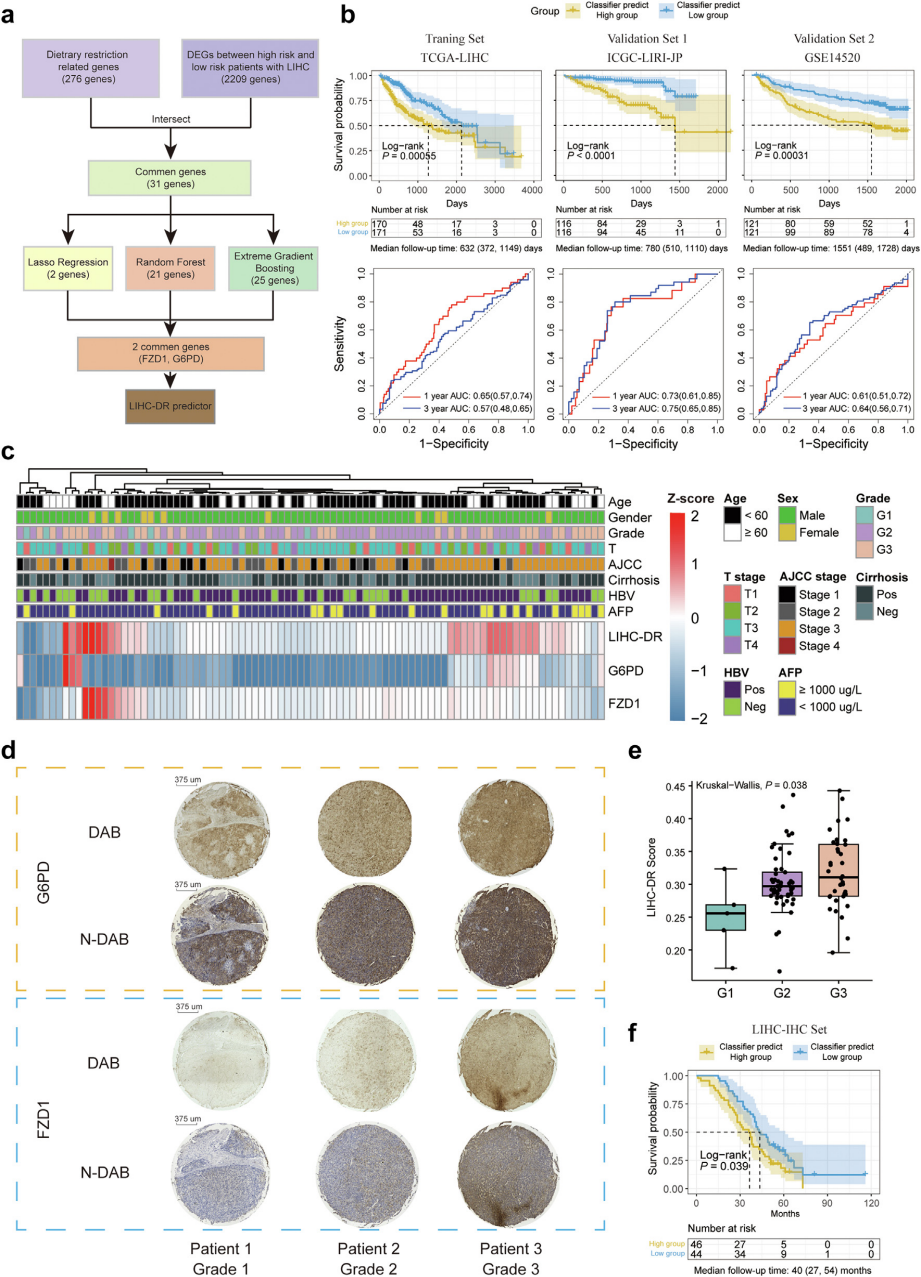
**DR genes are potential predictors of patient prognosis in liver cancer**. (a) Overall strategies for selecting two key genes in the DR signature to predict the OS of patients with LIHC in the TCGA. (b) Kaplan–Meier curves of OS (upper panel) and the receiver operating characteristic (ROC) curves of the LIHC-DR predictor score in predicting the OS (bottom panel) for patients with high and low LIHC-DR predictor scores in three published LIHC cohorts, including the training set (TCGA) and two validation datasets (ICGC-LIRI-JP and GSE14520). The shaded area refers to the 95% confidence band for survival curves. (c) Heatmap showing the quantitative results of the G6PD, FZD1 proteins, and their corresponding LIHC-DR predictor scores in IHC assays of 90 patients with LIHC. The annotation bar of the heatmap showing the baseline clinical characteristics of the 90 patients with LIHC. (d) Representative IHC graph of G6PD and FZD1 expression in three patients with LIHC. Tumor grades are indicated at the bottom. DAB, only DAB staining. N-DAB, nuclear, and DAB staining. Scale bar: 375 μm.(e) Boxplots comparing LIHC-DR predictor scores among different tumour grades of patients with LIHC. In Box-and-whisker plots, an outlier is defined as a data point located outside the whiskers that is outside 1.5 times the interquartile range above the upper quartile and below the lower quartile. (f) Kaplan–Meier curves of OS for patients with high and low LIHC-DR predictor scores in IHC assays of 90 patients with LIHC. The shaded area refers to the 95% confidence band for survival curves. For each survival analysis, the median (IQR) of the follow-up time is reported.

To develop a simplified DR predictor and identify the critical genes, we collected 3 LIHC cohorts (TCGA-LIHC, ICGC-LIRI-JP, and GSE14520) with a total of 815 patients to develop a Cox proportional hazard regression model and utilized three machine-learning algorithms to select the key genes. The TCGA-LIHC cohort was used as the training cohort, while the others served as external validation cohorts. In the TCGA-LIHC cohort, the LASSO, RFB, and XGBoost algorithms identified 2, 21, and 25 critical DR-related genes, respectively (Fig. 8a). Finally, two genes (frizzled class receptor 1, FZD1; glucose-6-phosphate dehydrogenase, G6PD) were extracted to construct the LIHC-DR predictor. We defined the LIHC-DR predictor as follows: LIHC-DR predictor = 0.001906 × (expression of FZD1) + 0.000974 × (expression of G6PD). The DEGs analysis between high-DR group and low-DR group of patients with LIHC revealed the FZD1 and G6PD were highly expressed in low-DR group (Figure S11c). Patients with higher LIHC-DR predictors (divided by the median of LIHC-DR predictors) exhibited a significantly worse prognosis in all three cohorts (Fig. 8b). The LIHC-DR predictor achieved an average AUC of approximately 0.65 for predicting 1-year and 3-year progressions.

To validate the predictive efficacy of the LIHC-DR predictor, we enrolled 90 patients with LIHC and conducted immunohistochemical (IHC) staining for FZD1 and G6PD (Fig. 8c and d). As expected, the LIHC-DR predictor could predict a LIHC-DR score that positively correlated to the tumour differentiation grade (Fig. 8e). More importantly, the LIHC-DR predictor could effectively categorize patients with LIHC who may experience a worse prognosis into the high-risk group in the IHC validation cohort, in which we observed significant differences in OS between the low-risk and high-risk groups of the patients with LIHC (*P* = 0.039, log-rank test, Fig. 8f).

Collectively, these results illustrated that DR-related molecular activities were correlated with the prognosis of specific cancer types, including LIHC. Moreover, the LIHC-DR predictor exhibits a significant correlation with clinical outcomes in diverse cohorts of patients with LIHC, suggesting that FZD1 and G6PD hold potential as prognostic biomarkers in LIHC.

## Discussion

DR is undoubtedly the most robust non-pharmacological intervention against induced and spontaneous cancers.^79^ Numerous studies have shown the ability of daily DR to delay neoplasia in multiple tissues and inhibit the growth of chemically induced and spontaneous tumours.^80^ DR-related molecular activities play a crucial role in mediating the anti-cancer effects of DR, while the comprehensive assessments of landscape of these activities and their associated biological features at pan-cancer level remain to be explored. In this study, we proposed an integrative approach for DR-related molecular activities quantification and uncovered the associations between these activities and tumour microenvironment, immune phenotypes, genomic features, and clinical prognosis in specific cancer types. Our findings establish a framework for enhancing the understanding of DR-related modulations in the tumour microenvironment. This framework could stimulate further investigations into DR-related mechanisms and clinical applications.

This study provides a comprehensive evaluation of DR-related molecular activities at the pan-cancer level by integrating DR-related genes and proposing a metric (DR score) to illustrate the DR-related modulations and molecular features in the tumour microenvironment. This multiparametric metric was constructed by DR-related genes, which were identified in genetic manipulation experiments and multiple microarray analyses across various preclinical animal models, indicating its potential for wide-ranging applicability that could be used as a criterion to represent the levels of DR-related molecular activities in diverse tissues. Moreover, this measurement can be utilized in data of single-cell transcriptome and ST, which could help to assess the levels of DR-related molecular activities in cells lacking detectable markers at single-cell level. With this measure index, we found the DR scores were lower in the tumour samples and were negatively correlated with the parameters of aggressive phenotypes, which aligns with previous literature that DR could repress carcinogenesis.^81^ Additionally, we found the DR-related molecular activities exhibited intercellular heterogeneity and tissue-preferential distribution, suggesting the critical contribution of cell origins and tissue types to the DR score.

Using a multi-omics approach, we discovered a close association between DR scores and tumour immunity. At single-cell level, we found the DR score could reflect the status of immune cells in tumour microenvironment, in which the immune cells in low DR group were highly involved in the cell–cell communication with tumour cells but may be inhibited from undergoing apoptosis. Consistently, the ST analysis revealed a similar result. As the contact between immune cells and tumour cells intensified, the DR scores of immune cells decreased significantly, while the interactions between immune cells and tumour cells increased. At the pan-cancer level, DR scores exhibited negative correlations with total immune cell infiltration levels, which supports previous findings indicating that DR can reduce inflammation levels.^82^ Moreover, the DR score showed a negative correlation with immunosuppression. In most cancer types, DR scores displayed negative correlations with Tregs infiltration, consistent with previous findings suggesting that DR can inhibit the migration of Tregs toward tumour cells.^83^ DR scores also showed negative correlations with PD-L1 expression in the majority of cancer types. The comparison between low-DR and high-DR groups at pan-cancer level revealed that patients with high levels of DR-related molecular activities were less likely to experience unfavourable outcomes which attributed the cause to severe immunosuppression. This finding is consistent with previous result that a high level of DR-related molecular activities can reverse immunosuppression.^84^

Most importantly, DR-related genes have significant clinical implications in patient prognosis and immunotherapy. Our pan-cancer analysis revealed that patients in the low-DR group exhibited severe immunosuppression but abundant immune infiltration, particularly with cytolytic T-cells. These results suggest that the DR score can help identify patients with specific immune characteristics that make them more susceptible to immunotherapy. Analysis of diverse ICB datasets at the single-cell and tissue level showed significant differences in DR scores between responders and non-responders, indicating the DR score can serve as a biomarker for predicting the response to ICB treatment in various cancer types. Furthermore, the DR score was a protectively independent risk predictor for OS and CSS at pan-cancer level, which was in line with the findings from several clinical trials, including breast cancer.^85^^,^^86^ To be more specific, we identified two hub genes from the DR-related genes using three machine-learning algorithms and used the genes to construct a promising predictor for predicting the prognosis of patients with LIHC. These genes, FZD1 and G6PD, demonstrate protumoral functions in hepatocellular carcinoma via various mechanisms.^87^^,^^88^ However, a comprehensive prognostic model combining these genes and important clinical prognostic indicators is still needed after gathering sufficient clinical information in future studies.

At last, our findings revealed a significant correlation between the DR scores and both genomic instability and genomic heterogeneity. The low DR scores strongly correlate with genomic alterations associated with aggressive phenotypes across diverse types of cancer. These results provide genomic insights into the inhibitory effects of DR on cancer at a pan-cancer level.

Our study has several limitations. Firstly, the GSVA-based strategy used to define DR-related molecular activities relies on the gene signature composition, which may require updates as new molecules associated with DR are discovered to enhance technical novelty. Secondly, while GSVA is more stable than other algorithms for assessing gene set activity in single-cell transcriptomes,^89^ high dropout rates could impact the accuracy of DR scores in evaluating the levels of DR-related molecular activities. Thirdly, the associations of DR-related molecular activities with immunotherapy responses need further validation in larger samples, as this study included a relatively limited number of immunotherapy treatment cohorts. Ultimately, more experimental validations are required to comprehensively interpret the DR score.

However, our findings are based on observational data without randomization. There could be unmeasured confounding. The hazards may not distribute evenly over time, therefore, the HR of DR score observed in the present study could be affected by built-in selection bias. Future studies are needed to further validate these findings. Additionally, we found that DR scores were the independent protective factors in 33 types of cancer. However, some of these results may be disturbed by sparse data bias, especially in cancer species with limited data.

In conclusion, based on computational metrics of DR-related molecular activities, our findings provide a framework for a better understanding of DR-related molecular and biological features in the tumour microenvironment at pan-cancer level. Furthermore, our work has the potential to advance the development of DR-related mechanism studies and personalized therapeutic strategies in clinical oncology.

## Contributors

Guarantors of integrity of the entire study, XYS, WJX, YL, WZ, ZYC, KWL, WJY, JYL, WPP, ML; study concepts/study design or data acquisition or data analysis/interpretation, XYS, WJX, YL, ML; manuscript drafting or manuscript revision for important intellectual content, XYS, ML; approval of final version of submitted manuscript, all authors; agrees to ensure any questions related to the work are appropriately resolved, all authors; literature research, XYS, WJX, YL, ML; clinical studies, XYS, WJX, YL, WZ, ZYC, KWL, WJY, JYL, WPP, ML; experimental studies, XYS, WJX, YL, ML; statistical analysis, XYS, ML; and manuscript editing, XYS, WJX, YL, ML. XYS, WJX, YL, and ML have verified the underlying data used in this study.

## Data sharing statement

The multi-omics data and clinical information of the patients, or patients with immunotherapy, used in this study were described in the method section “Data and resources” and were all public available. The resources and tools used in our analyses were described in each method section.

## Declaration of interests

The authors declare that they have no conflict of interest.

## Appendix A Supplementary data

Supplementary Figures

Antibody validation file
