# Supplementary material for: An integrative pan-cancer analysis of the molecular characteristics of dietary restriction in tumour microenvironment
Source: eBioMedicine. 2024 Mar 19;102:105078. doi: 10.1016/j.ebiom.2024.105078 (PMC10965464; doi:10.1016/j.ebiom.2024.105078)
Supplement: Supplementary Figures [file mmc2.pdf]

Supplementary Material

Supplementary Figures

Contents	Pages
**Figure S1.** Go enrichment analysis of DR genes and the DR scores in CCLE cell lines.	2
**Figure S2.** The association between DR scores and obesity.	3
**Figure S3.** Heterogeneity of DR-related molecular activities in different cancer types.	4
**Figure S4.** Transcription-level PD-1/PDL1 activity among immune cells in different DR group.	5
**Figure S5.** The summary of the mutation events in high-DR and low-DR groups.	6
**Figure S6.** The associations between DR scores and arm-level CNV events.	7
**Figure S7.** Identifying the clinical relevance of DR scores in various cancer types.	8
**Figure S8.** Consensus clustering identified two similar clusters in ICGC-LIRI-JP dataset.	9
**Figure S9.** Cancer type-specific associations of DR scores with immunotherapy sensitivity indexes.	10
**Figure S10.** The correlation between the DR scores and the drug sensitivity of chemotherapy and targeted therapy agents.	11
**Figure S11.** Cancer type-specific associations of DR scores with survival.	12

Figure S1

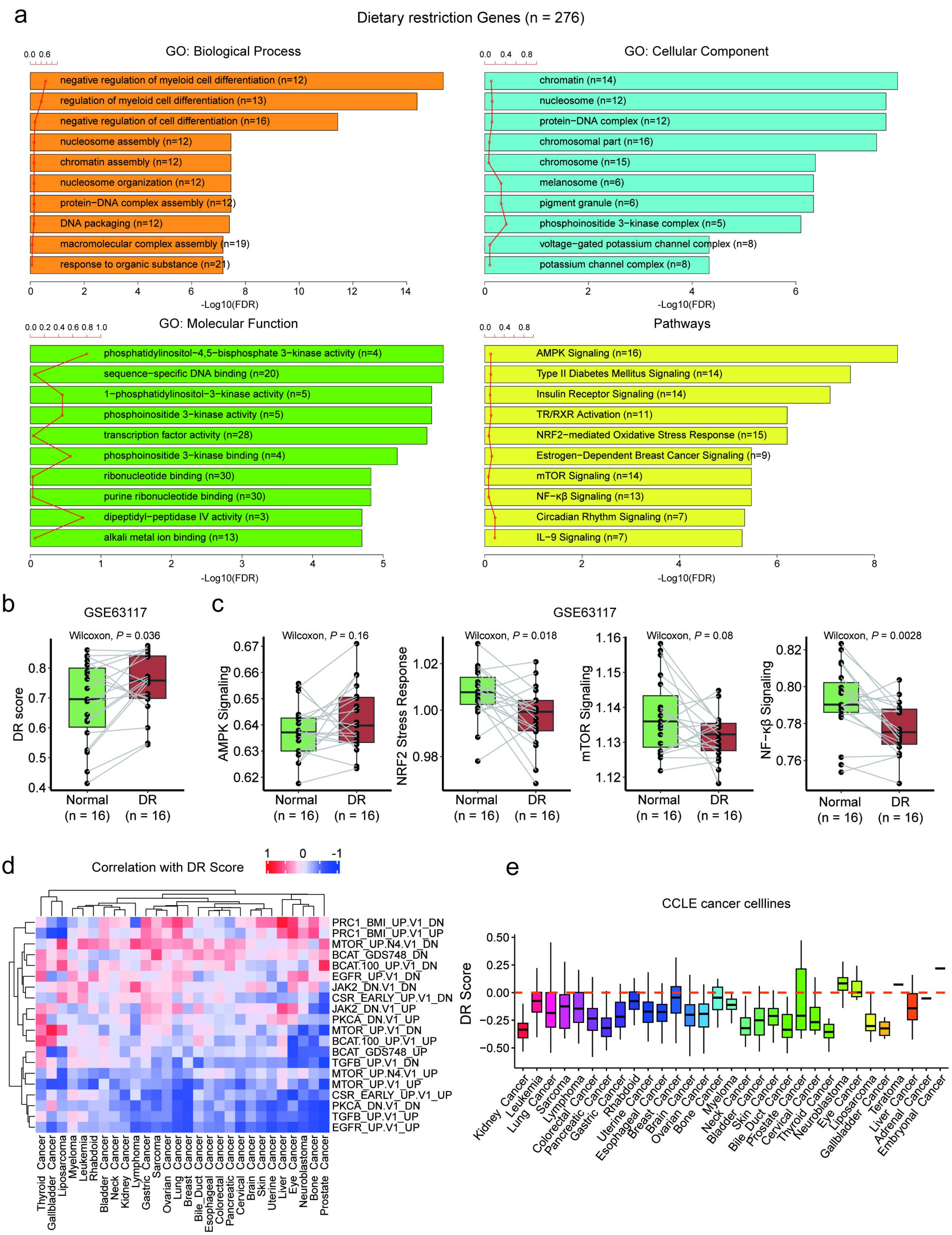
a
Dietary restriction Genes (n = 276)
GO: Biological Process
negative regulation of myeloid cell differentiation (n=12)
regulation of myeloid cell differentiation (n=13)
negative regulation of cell differentiation (n=16)
nucleosome assembly (n=12)
chromatin assembly (n=12)
nucleosome organization (n=12)
protein−DNA complex assembly (n=12)
DNA packaging (n=12)
macromolecular complex assembly (n=19)
response to organic substance (n=21)
-Log10(FDR)
GO: Cellular Component
chromatin (n=14)
nucleosome (n=12)
protein−DNA complex (n=12)
chromosomal part (n=16)
chromosome (n=15)
melanosome (n=6)
pigment granule (n=6)
phosphoinositide 3−kinase complex (n=5)
voltage−gated potassium channel complex (n=8)
potassium channel complex (n=8)
GO: Molecular Function
phosphatidylinositol−4,5−bisphosphate 3−kinase activity (n=4)
sequence−specific DNA binding (n=20)
1−phosphatidylinositol−3−kinase activity (n=5)
phosphoinositide 3−kinase activity (n=5)
transcription factor activity (n=28)
phosphoinositide 3−kinase binding (n=4)
ribonucleotide binding (n=30)
purine ribonucleotide binding (n=30)
dipeptidyl−peptidase IV activity (n=3)
alkali metal ion binding (n=13)
Pathways
AMPK Signaling (n=16)
Type II Diabetes Mellitus Signaling (n=14)
Insulin Receptor Signaling (n=14)
TR/RXR Activation (n=11)
NRF2−mediated Oxidative Stress Response (n=15)
Estrogen−Dependent Breast Cancer Signaling (n=9)
mTOR Signaling (n=14)
NF−κβ Signaling (n=13)
Circadian Rhythm Signaling (n=7)
IL−9 Signaling (n=7)
b
GSE63117
Wilcoxon, P = 0.036
DR score
Normal (n = 16)
DR (n = 16)
c
GSE63117
Wilcoxon, P = 0.16
AMPK Signaling
Wilcoxon, P = 0.018
NRF2 Stress Response
Wilcoxon, P = 0.08
mTOR Signaling
Wilcoxon, P = 0.0028
NF−κβ Signaling
d
Correlation with DR Score
PRC1_BMI_UP.V1_DN
PRC1_BMI_UP.V1_UP
MTOR_UP.N4.V1_DN
BCAT_GDS748_DN
BCAT.100_UP.V1_DN
EGFR_UP.V1_DN
JAK2_DN.V1_DN
CSR_EARLY_UP.V1_DN
JAK2_DN.V1_UP
PKCA_DN.V1_UP
MTOR_UP.V1_DN
BCAT.100_UP.V1_UP
BCAT_GDS748_UP
TGFB_UP.V1_DN
MTOR_UP.N4.V1_UP
MTOR_UP.V1_UP
CSR_EARLY_UP.V1_UP
PKCA_DN.V1_DN
TGFB_UP.V1_UP
EGFR_UP.V1_UP
e
CCLE cancer celllines
DR Score
Kidney Cancer
Leukemia
Lung Cancer
Sarcoma
Lymphoma
Colorectal Cancer
Pancreatic Cancer
Gastric Cancer
Rhabdoid
Uterine Cancer
Esophageal Cancer
Breast Cancer
Brain Cancer
Ovarian Cancer
Bone Cancer
Myeloma
Neck Cancer
Bladder Cancer
Skin Cancer
Bile_Duct Cancer
Prostate Cancer
Cervical Cancer
Thyroid Cancer
Neuroblastoma
Eye Cancer
Liposarcoma
Gallbladder Cancer
Teratoma
Liver Cancer
Adrenal Cancer
Embryonal Cancer

Figure S2

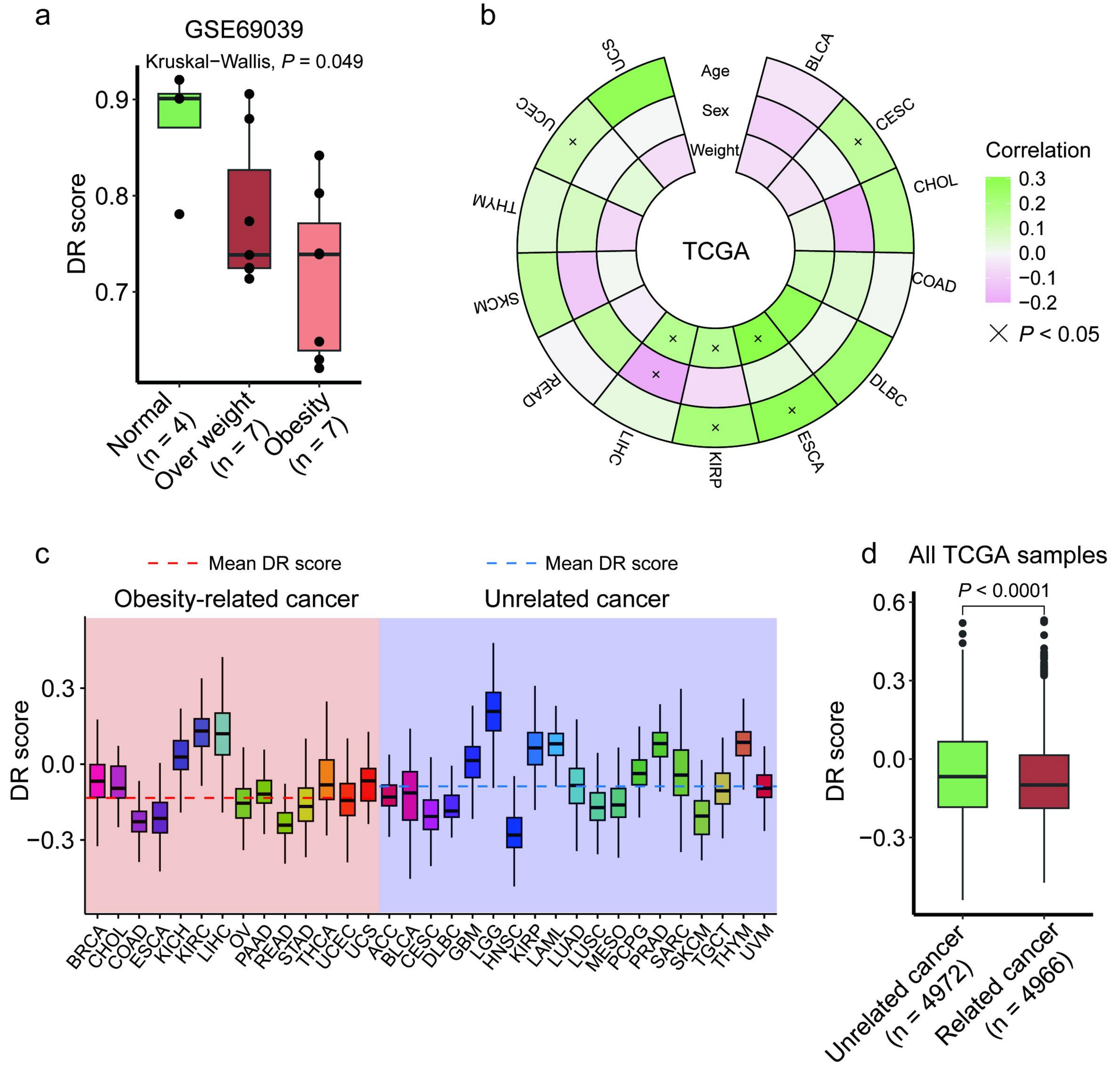
a
GSE69039
Kruskal−Wallis, P = 0.049
DR score
0.9
0.8
0.7
Normal (n = 4)
Over weight (n = 7)
Obesity (n = 7)
b
TCGA
Age
Sex
Weight
UCS
BLCA
CESC
CHOL
COAD
DLBC
ESCA
KIRP
LIHC
READ
SKCM
THYM
UCEC
Correlation
0.3
0.2
0.1
0.0
−0.1
−0.2
P < 0.05
c
Mean DR score
Mean DR score
Obesity-related cancer
Unrelated cancer
DR score
0.3
0.0
−0.3
BRCA
CHOL
COAD
ESCA
KICH
KIRC
LIHC
OV
PAAD
READ
STAD
THCA
UCEC
UCS
ACC
BLCA
CESC
DLBC
GBM
LGG
HNSC
KIRP
LAML
LUAD
LUSC
MESO
PCPG
PRAD
SARC
SKCM
TGCT
THYM
UVM
d
All TCGA samples
P < 0.0001
DR score
0.6
0.3
0.0
−0.3
Unrelated cancer (n = 4972)
Related cancer (n = 4966)

Figure S3

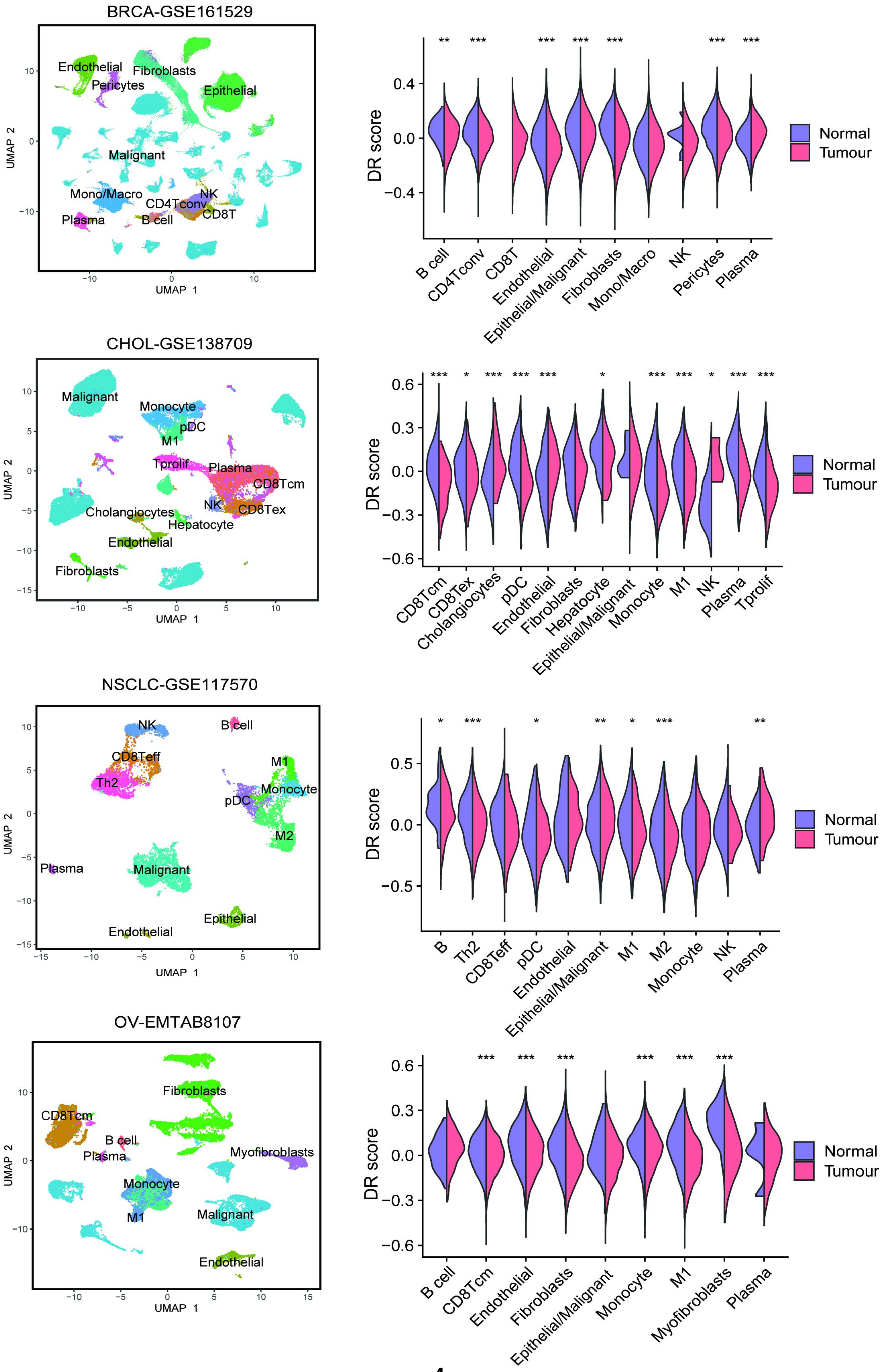

BRCA-GSE161529
Endothelial
Fibroblasts
Pericytes
Epithelial
Malignant
Mono/Macro
NK
CD4Tconv
CD8T
Plasma
B cell
UMAP 2
UMAP 1
10
0
−10
**
***
***
***
***
***
***
0.4
0.0
−0.4
DR score
B cell
CD4Tconv
CD8T
Endothelial
Epithelial/Malignant
Fibroblasts
Mono/Macro
NK
Pericytes
Plasma
Normal
Tumour
CHOL-GSE138709
Malignant
Monocyte
pDC
M1
Tprolif
Plasma
CD8Tcm
NK
CD8Tex
Cholangiocytes
Hepatocyte
Endothelial
Fibroblasts
UMAP 2
UMAP 1
10
5
0
−5
−10
−15
***
*
***
***
***
*
***
***
*
***
***
0.6
0.3
0.0
−0.3
−0.6
DR score
CD8Tcm
CD8Tex
Cholangiocytes
pDC
Endothelial
Fibroblasts
Hepatocyte
Epithelial/Malignant
Monocyte
M1
NK
Plasma
Tprolif
Normal
Tumour
NSCLC-GSE117570
NK
B cell
CD8Teff
M1
Th2
Monocyte
pDC
M2
Plasma
Malignant
Epithelial
Endothelial
UMAP 2
UMAP 1
10
5
0
−5
−10
−15
*
***
*
**
*
***
**
0.5
0.0
−0.5
DR score
B
Th2
CD8Teff
pDC
Endothelial
Epithelial/Malignant
M1
M2
Monocyte
NK
Plasma
Normal
Tumour
OV-EMTAB8107
Fibroblasts
CD8Tcm
B cell
Plasma
Myofibroblasts
Monocyte
M1
Malignant
Endothelial
UMAP 2
UMAP 1
10
0
−10
−5
5
15
***
***
***
***
***
***
0.6
0.3
0.0
−0.3
−0.6
DR score
B cell
CD8Tcm
Endothelial
Fibroblasts
Epithelial/Malignant
Monocyte
M1
Myofibroblasts
Plasma
Normal
Tumour

Figure S4

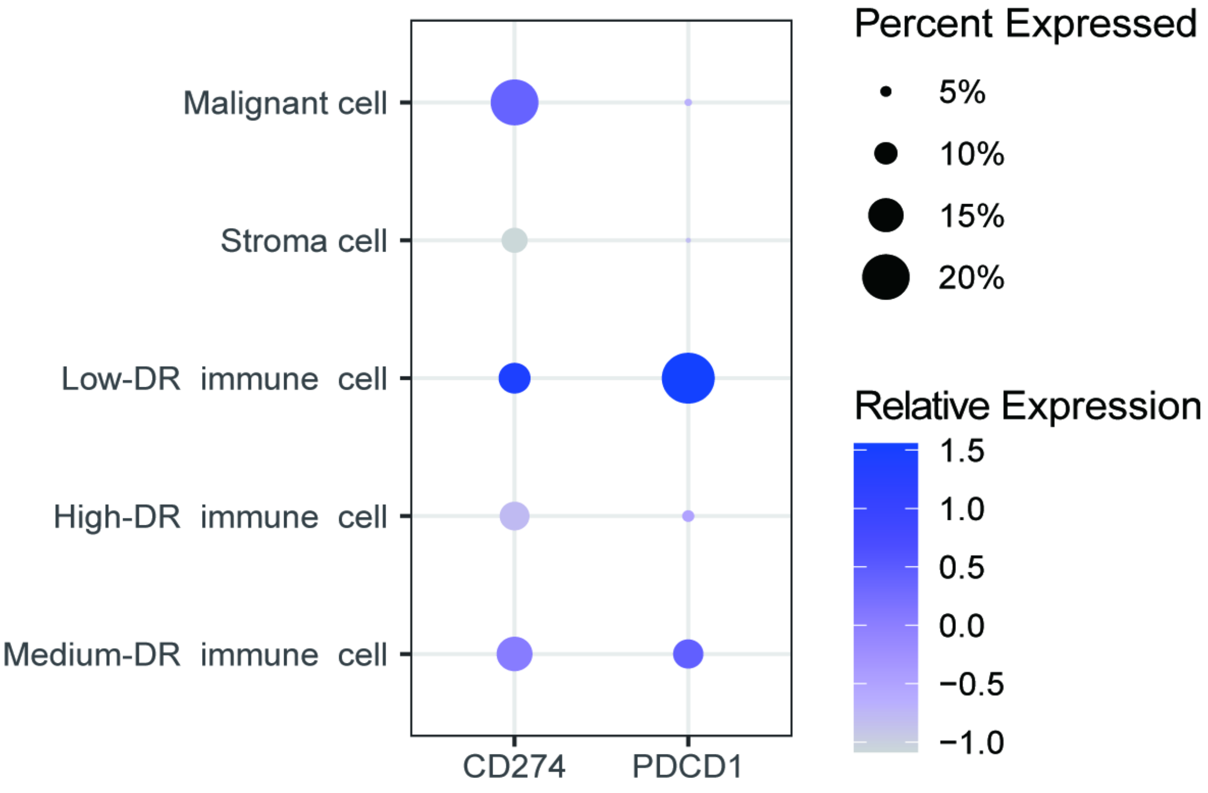
Malignant cell
Stroma cell
Low-DR immune cell
High-DR immune cell
Medium-DR immune cell
CD274
PDCD1
Percent Expressed
5%
10%
15%
20%
Relative Expression
1.5
1.0
0.5
0.0
−0.5
−1.0

Figure S5

a

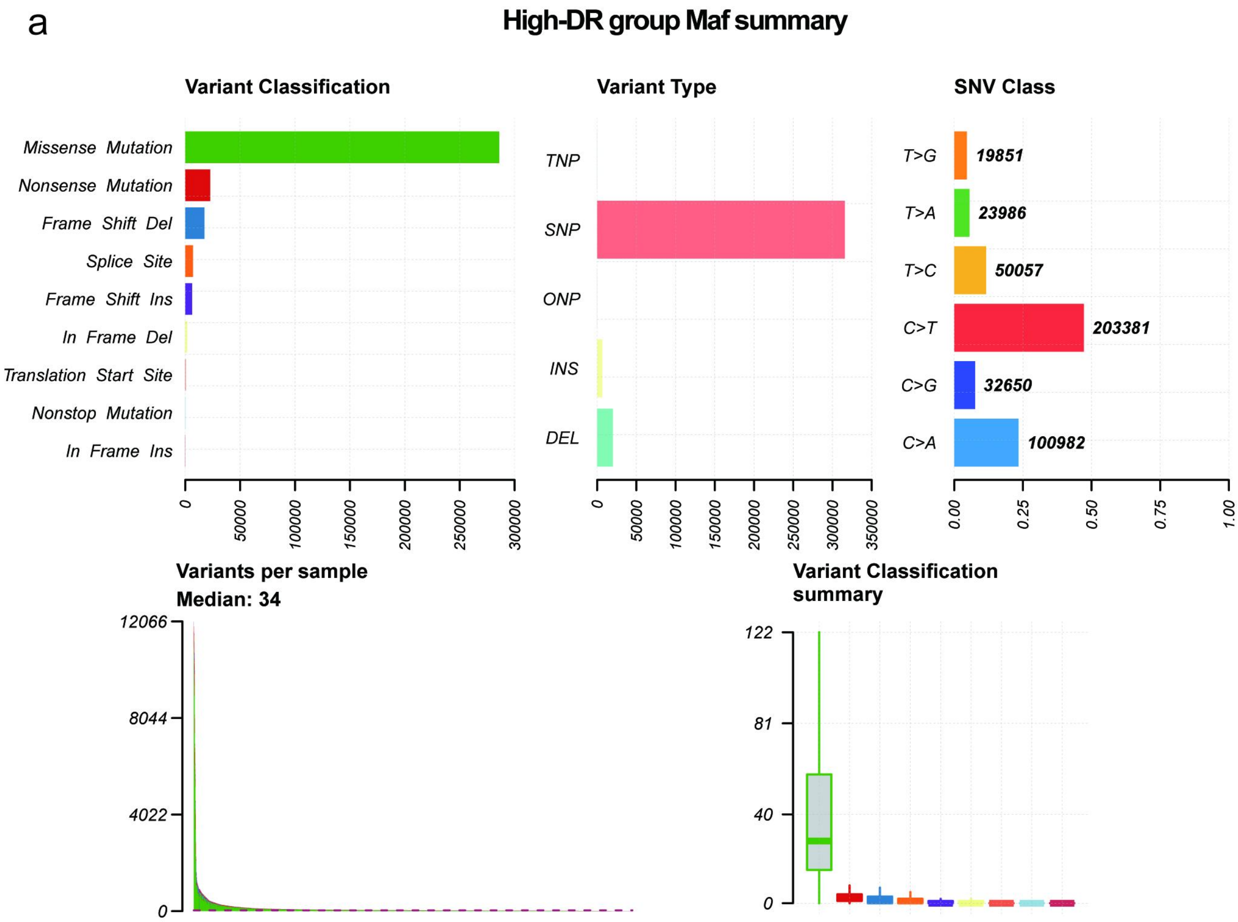
High-DR group Maf summary
Variant Classification
Missense Mutation
Nonsense Mutation
Frame Shift Del
Splice Site
Frame Shift Ins
In Frame Del
Translation Start Site
Nonstop Mutation
In Frame Ins
Variant Type
TNP
SNP
ONP
INS
DEL
SNV Class
T>G 19851
T>A 23986
T>C 50057
C>T 203381
C>G 32650
C>A 100982
Variants per sample
Median: 34
Variant Classification summary

b

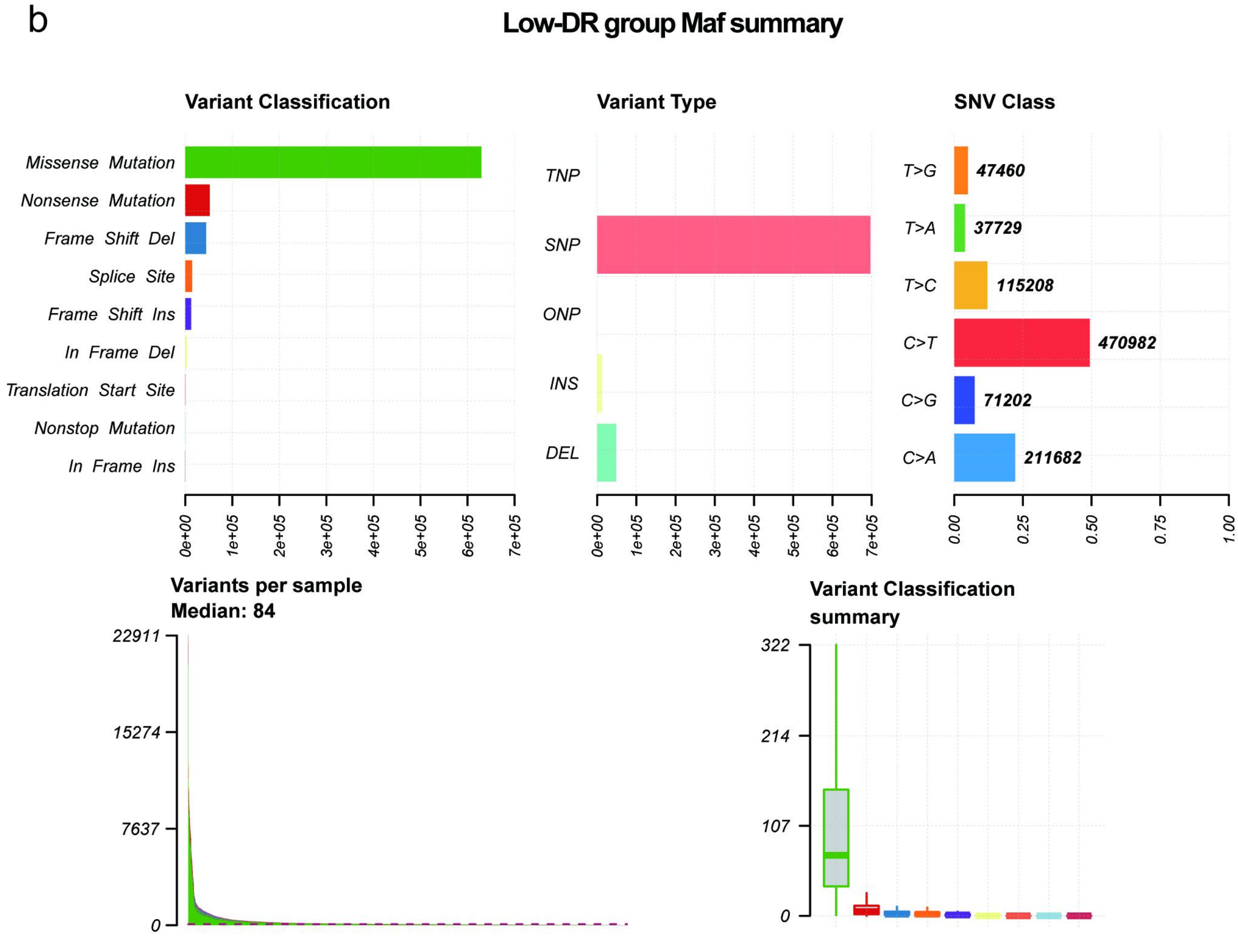
Low-DR group Maf summary
Variant Classification
Missense Mutation
Nonsense Mutation
Frame Shift Del
Splice Site
Frame Shift Ins
In Frame Del
Translation Start Site
Nonstop Mutation
In Frame Ins
Variant Type
TNP
SNP
ONP
INS
DEL
SNV Class
T>G 47460
T>A 37729
T>C 115208
C>T 470982
C>G 71202
C>A 211682
Variants per sample
Median: 84
Variant Classification summary

Figure S6

a

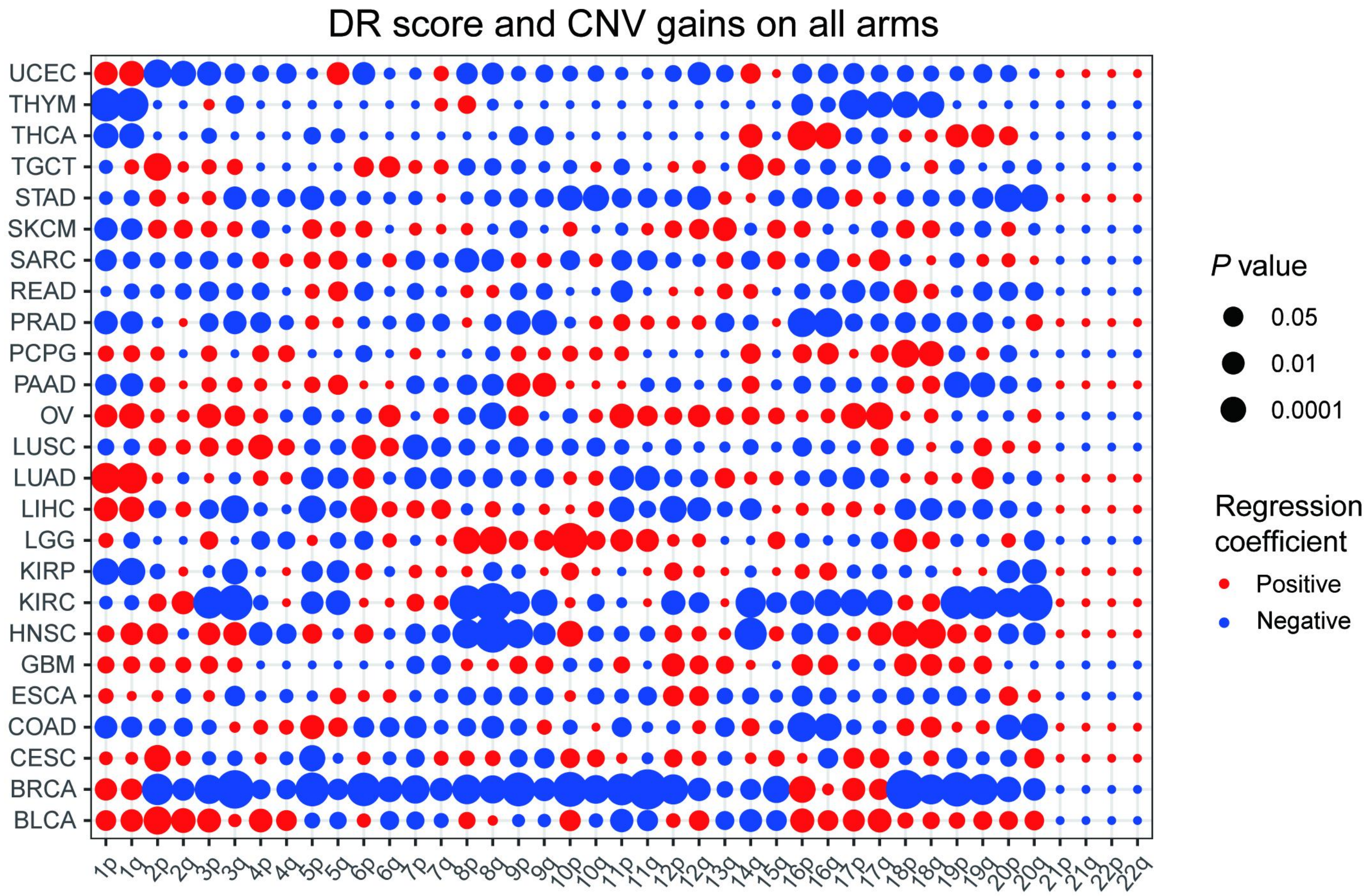
DR score and CNV gains on all arms
UCEC
THYM
THCA
TGCT
STAD
SKCM
SARC
READ
PRAD
PCPG
PAAD
OV
LUSC
LUAD
LIHC
LGG
KIRP
KIRC
HNSC
GBM
ESCA
COAD
CESC
BRCA
BLCA
1p 1q 2p 2q 3p 3q 4p 4q 5p 5q 6p 6q 7p 7q 8p 8q 9p 9q 10p 10q 11p 11q 12p 12q 13q 14q 15q 16p 16q 17p 17q 18p 18q 19p 19q 20p 20q 21p 21q 22p 22q
P value
0.05
0.01
0.0001
Regression coefficient
Positive
Negative

b

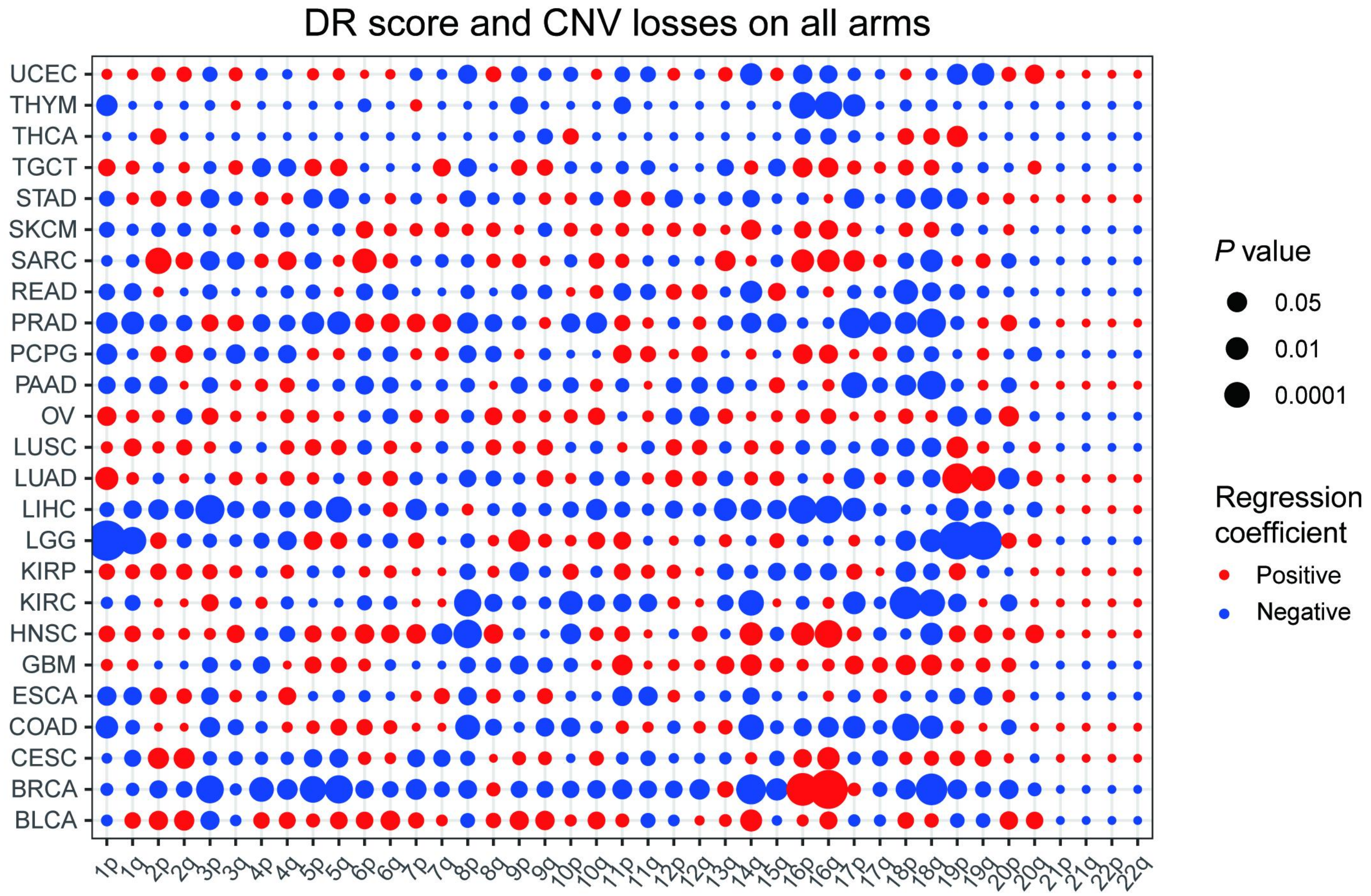
DR score and CNV losses on all arms
UCEC
THYM
THCA
TGCT
STAD
SKCM
SARC
READ
PRAD
PCPG
PAAD
OV
LUSC
LUAD
LIHC
LGG
KIRP
KIRC
HNSC
GBM
ESCA
COAD
CESC
BRCA
BLCA
1p 1q 2p 2q 3p 3q 4p 4q 5p 5q 6p 6q 7p 7q 8p 8q 9p 9q 10p 10q 11p 11q 12p 12q 13q 14q 15q 16p 16q 17p 17q 18p 18q 19p 19q 20p 20q 21p 21q 22p 22q
P value
0.05
0.01
0.0001
Regression coefficient
Positive
Negative

Figure S7

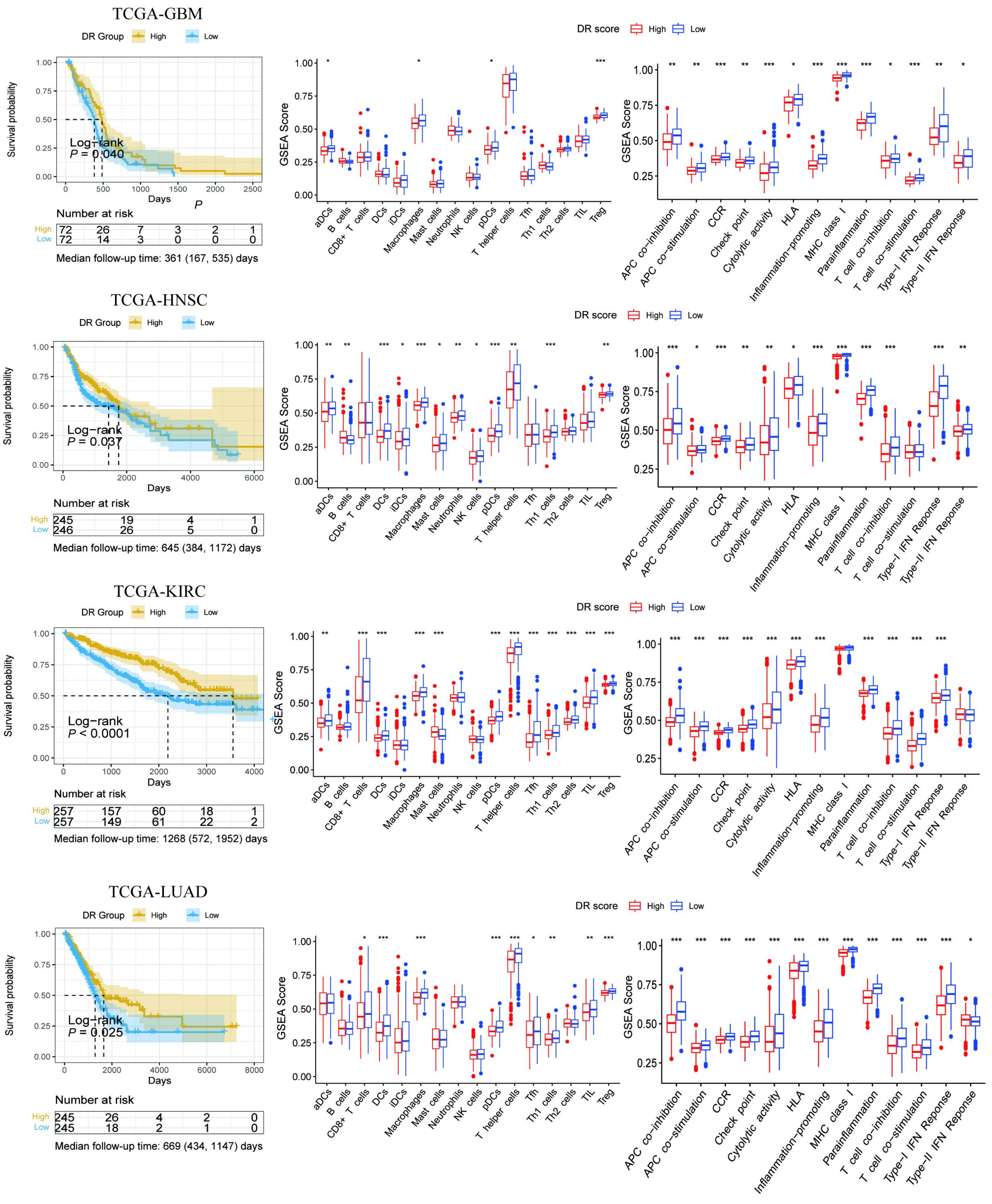
TCGA-GBM
DR Group High Low
Survival probability
Log−rank
P = 0.040
Days
Number at risk
High 72 26 7 3 2 1
Low 72 14 3 0 0 0
Median follow-up time: 361 (167, 535) days
DR score High Low
GSEA Score
aDCs
B cells
CD8+ T cells
DCs
iDCs
Macrophages
Mast cells
Neutrophils
NK cells
pDCs
T helper cells
Tfh
Th1 cells
Th2 cells
TIL
Treg
APC co−inhibition
APC co−stimulation
CCR
Check point
Cytolytic activity
HLA
Inflammation−promoting
MHC class I
Parainflammation
T cell co−inhibition
T cell co−stimulation
Type−I IFN_Reponse
Type−II IFN Reponse
TCGA-HNSC
Log−rank
P = 0.037
Number at risk
High 245 19 4 1
Low 246 26 5 0
Median follow-up time: 645 (384, 1172) days
TCGA-KIRC
Log−rank
P < 0.0001
Number at risk
High 257 157 60 18 1
Low 257 149 61 22 2
Median follow-up time: 1268 (572, 1952) days
TCGA-LUAD
Log−rank
P = 0.025
Number at risk
High 245 26 4 2 0
Low 245 18 2 1 0
Median follow-up time: 669 (434, 1147) days

Figure S8

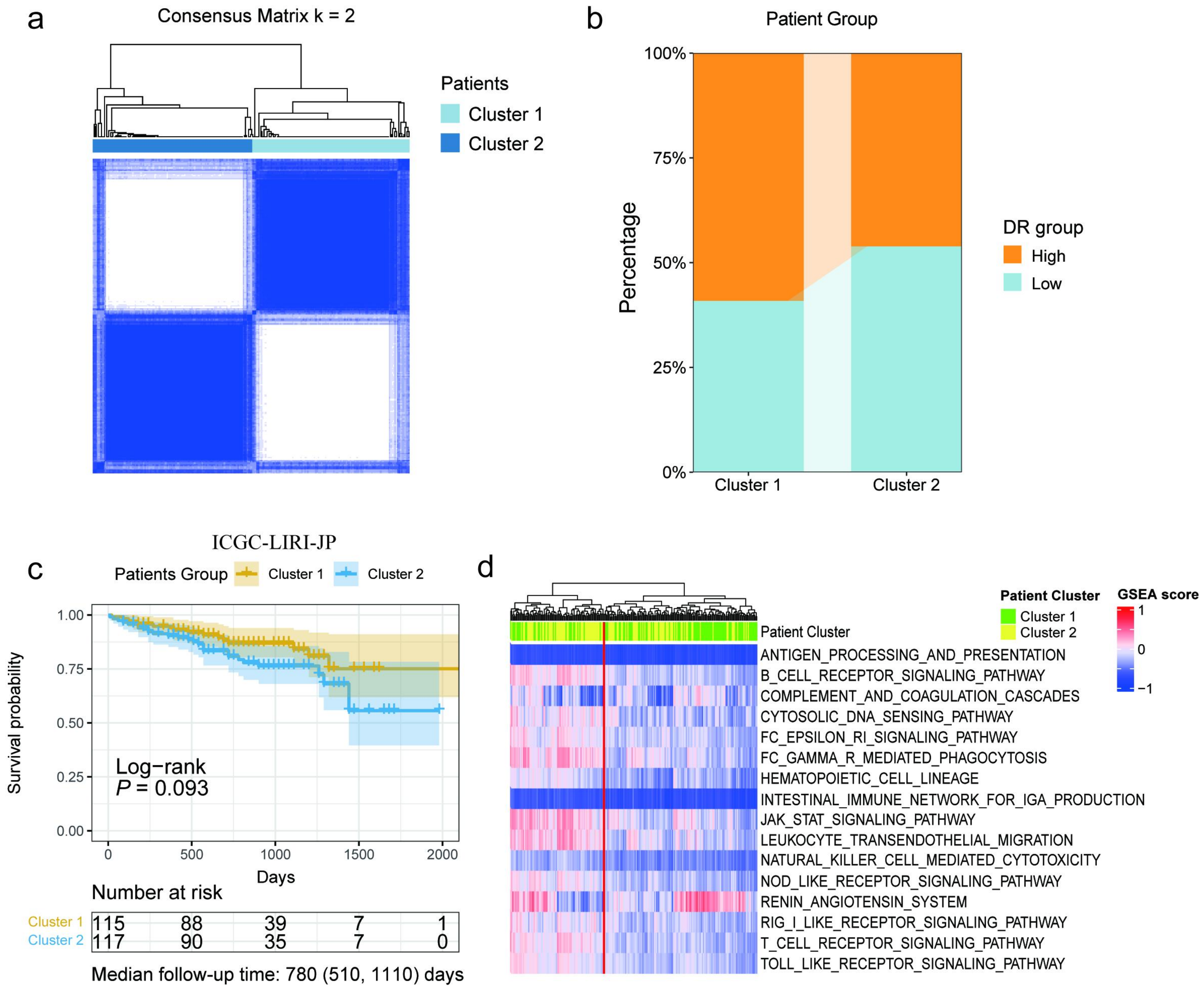
a
Consensus Matrix k = 2
Patients
Cluster 1
Cluster 2
b
Patient Group
100%
75%
50%
25%
0%
Percentage
Cluster 1
Cluster 2
DR group
High
Low
ICGC-LIRI-JP
c
Patients Group
Cluster 1
Cluster 2
1.00
0.75
0.50
0.25
0.00
Survival probability
Log−rank
P = 0.093
0
500
1000
1500
2000
Days
Number at risk
Cluster 1 115 88 39 7 1
Cluster 2 117 90 35 7 0
Median follow-up time: 780 (510, 1110) days
d
Patient Cluster
Cluster 1
Cluster 2
GSEA score
1
0
−1
Patient Cluster
ANTIGEN_PROCESSING_AND_PRESENTATION
B_CELL_RECEPTOR_SIGNALING_PATHWAY
COMPLEMENT_AND_COAGULATION_CASCADES
CYTOSOLIC_DNA_SENSING_PATHWAY
FC_EPSILON_RI_SIGNALING_PATHWAY
FC_GAMMA_R_MEDIATED_PHAGOCYTOSIS
HEMATOPOIETIC_CELL_LINEAGE
INTESTINAL_IMMUNE_NETWORK_FOR_IGA_PRODUCTION
JAK_STAT_SIGNALING_PATHWAY
LEUKOCYTE_TRANSENDOTHELIAL_MIGRATION
NATURAL_KILLER_CELL_MEDIATED_CYTOTOXICITY
NOD_LIKE_RECEPTOR_SIGNALING_PATHWAY
RENIN_ANGIOTENSIN_SYSTEM
RIG_I_LIKE_RECEPTOR_SIGNALING_PATHWAY
T_CELL_RECEPTOR_SIGNALING_PATHWAY
TOLL_LIKE_RECEPTOR_SIGNALING_PATHWAY

Figure S9

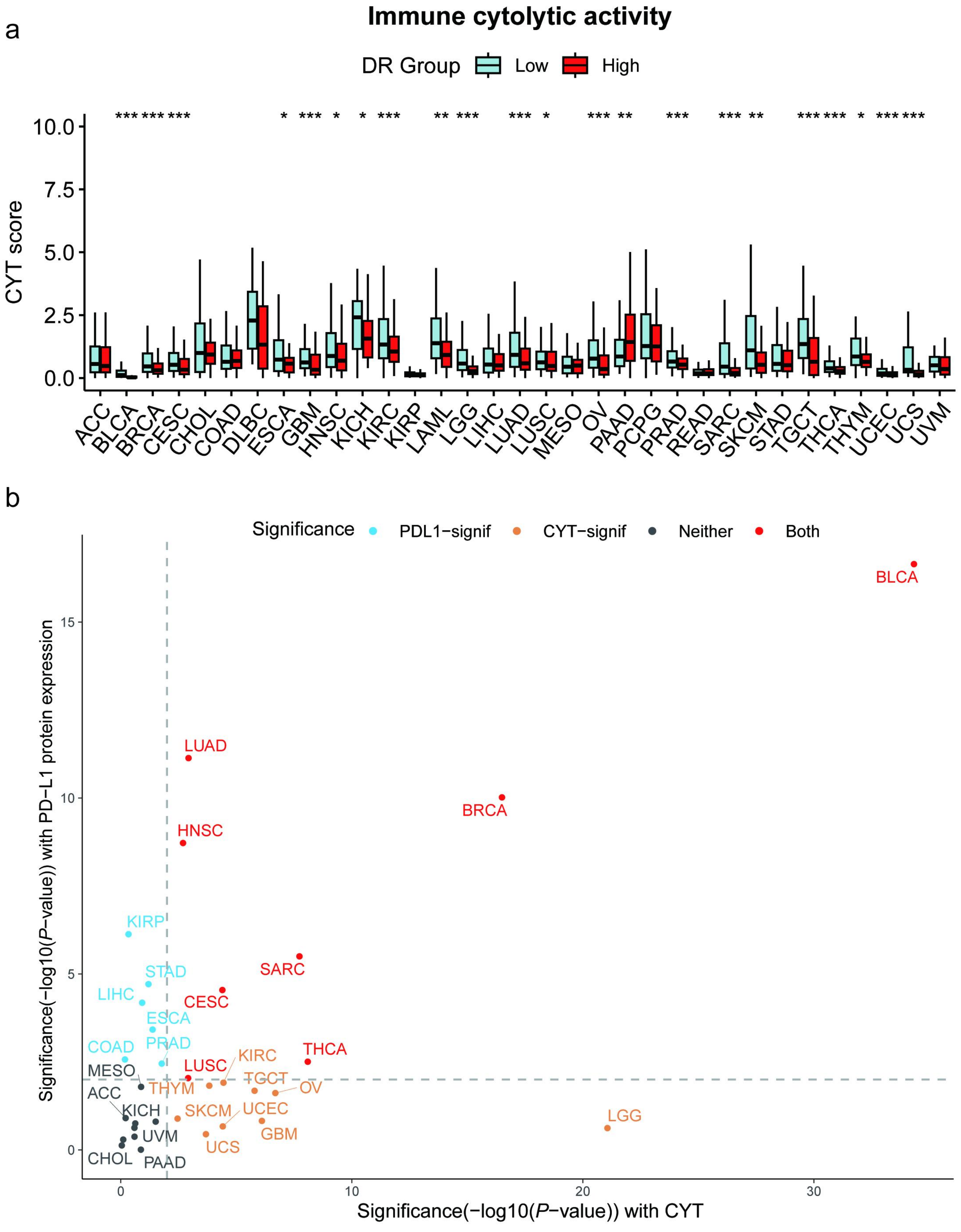
a
Immune cytolytic activity
DR Group Low High
CYT score
*** *** *** * *** * * *** ** *** *** * *** ** *** *** ** *** *** * *** ***
10.0
7.5
5.0
2.5
0.0
ACC BLCA BRCA CESC CHOL COAD DLBC ESCA GBM HNSC KICH KIRC KIRP LAML LGG LIHC LUAD LUSC MESO OV PAAD PCPG PRAD READ SARC SKCM STAD TGCT THCA THYM UCEC UCS UVM
b
Significance PDL1−signif CYT−signif Neither Both
Significance(−log10(P−value)) with PD−L1 protein expression
Significance(−log10(P−value)) with CYT
BLCA
LUAD
BRCA
HNSC
KIRP
STAD
SARC
LIHC
CESC
ESCA
COAD
PRAD
KIRC
THCA
MESO
LUSC
TGCT
THYM
OV
ACC
KICH
SKCM
UCEC
LGG
UVM
GBM
UCS
CHOL
PAAD
0
5
10
15
0
10
20
30

Figure S10

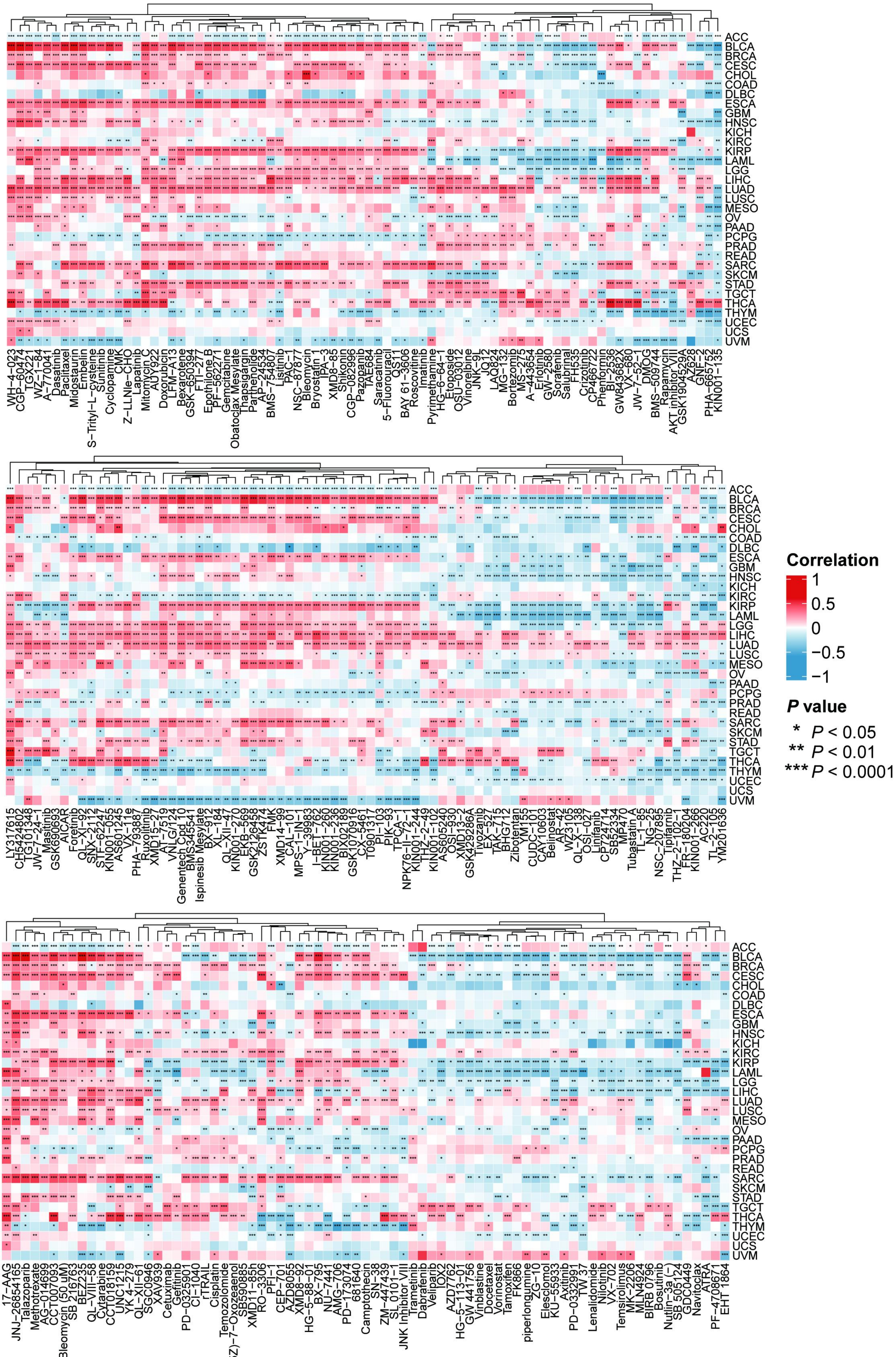
ACC
BLCA
BRCA
CESC
CHOL
COAD
DLBC
ESCA
GBM
HNSC
KICH
KIRC
KIRP
LAML
LGG
LIHC
LUAD
LUSC
MESO
OV
PAAD
PCPG
PRAD
READ
SARC
SKCM
STAD
TGCT
THCA
THYM
UCEC
UCS
UVM
Correlation
1
0.5
0
−0.5
−1
P value
* P < 0.05
** P < 0.01
*** P < 0.0001

Figure S11

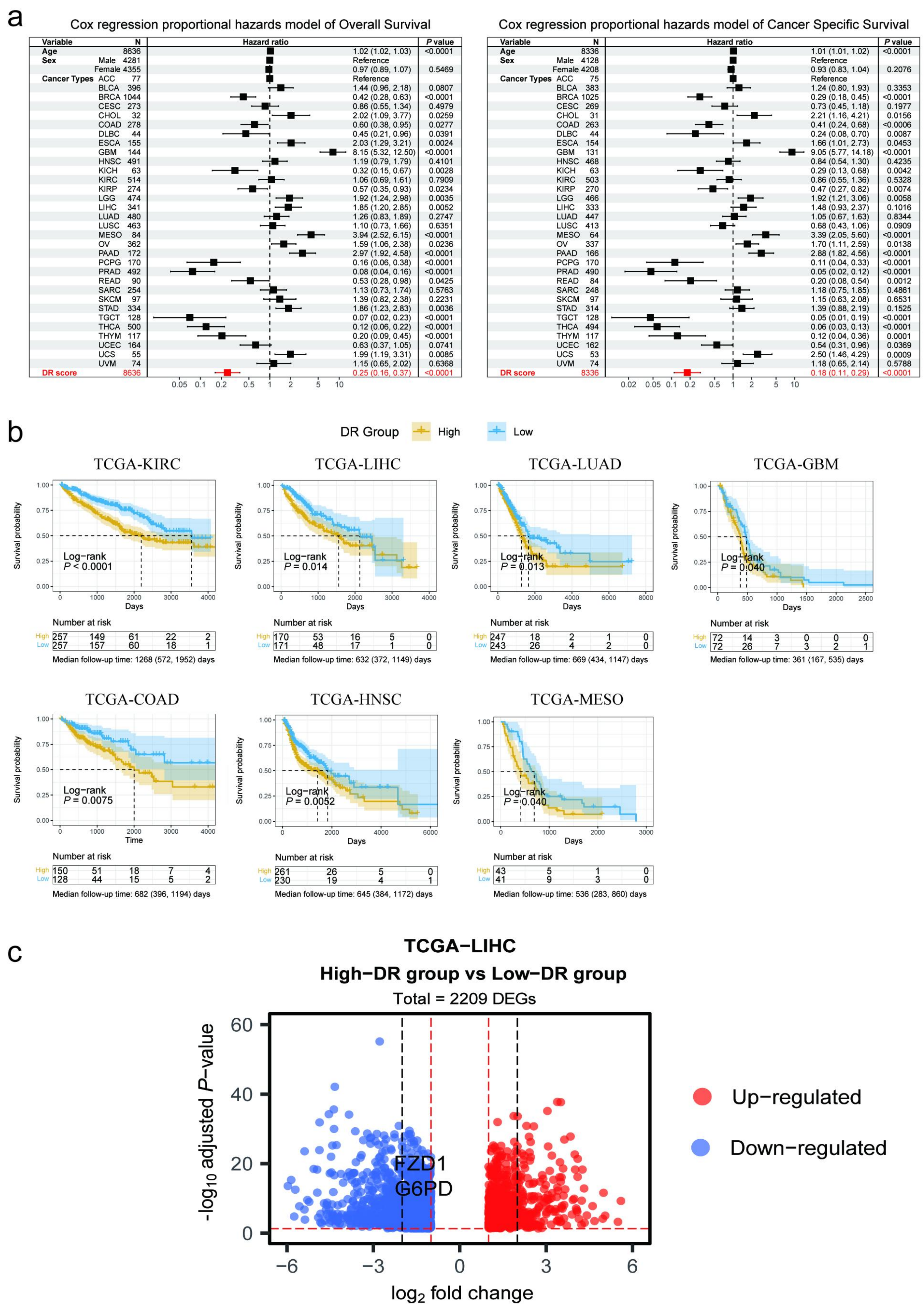
a
Cox regression proportional hazards model of Overall Survival
Variable N Hazard ratio P value
Age 8636 1.02 (1.02, 1.03) <0.0001
Sex Male 4281 Reference
Female 4355 0.97 (0.89, 1.07) 0.5469
Cancer Types ACC 77 Reference
BLCA 396 1.44 (0.96, 2.18) 0.0807
BRCA 1044 0.42 (0.28, 0.63) <0.0001
CESC 273 0.86 (0.55, 1.34) 0.4979
CHOL 32 2.02 (1.09, 3.77) 0.0259
COAD 278 0.60 (0.38, 0.95) 0.0277
DLBC 44 0.45 (0.21, 0.96) 0.0391
ESCA 155 2.03 (1.29, 3.21) 0.0024
GBM 144 8.15 (5.32, 12.50) <0.0001
HNSC 491 1.19 (0.79, 1.79) 0.4101
KICH 63 0.32 (0.15, 0.67) 0.0028
KIRC 514 1.06 (0.69, 1.61) 0.7909
KIRP 274 0.57 (0.35, 0.93) 0.0234
LGG 474 1.92 (1.24, 2.98) 0.0035
LIHC 341 1.85 (1.20, 2.85) 0.0052
LUAD 480 1.26 (0.83, 1.89) 0.2747
LUSC 463 1.10 (0.73, 1.66) 0.6351
MESO 84 3.94 (2.52, 6.15) <0.0001
OV 362 1.59 (1.06, 2.38) 0.0236
PAAD 172 2.97 (1.92, 4.58) <0.0001
PCPG 170 0.16 (0.06, 0.38) <0.0001
PRAD 492 0.08 (0.04, 0.16) <0.0001
READ 90 0.53 (0.28, 0.98) 0.0425
SARC 254 1.13 (0.73, 1.74) 0.5763
SKCM 97 1.39 (0.82, 2.38) 0.2231
STAD 334 1.86 (1.23, 2.83) 0.0036
TGCT 128 0.07 (0.02, 0.23) <0.0001
THCA 500 0.12 (0.06, 0.22) <0.0001
THYM 117 0.20 (0.09, 0.45) <0.0001
UCEC 164 0.63 (0.37, 1.05) 0.0741
UCS 55 1.99 (1.19, 3.31) 0.0085
UVM 74 1.15 (0.65, 2.02) 0.6368
DR score 8636 0.25 (0.16, 0.37) <0.0001
0.05 0.1 0.2 0.5 1 2 5 10
Cox regression proportional hazards model of Cancer Specific Survival
Variable N Hazard ratio P value
Age 8336 1.01 (1.01, 1.02) <0.0001
Sex Male 4128 Reference
Female 4208 0.93 (0.83, 1.04) 0.2076
Cancer Types ACC 75 Reference
BLCA 383 1.24 (0.80, 1.93) 0.3353
BRCA 1025 0.29 (0.18, 0.45) <0.0001
CESC 269 0.73 (0.45, 1.18) 0.1977
CHOL 31 2.21 (1.16, 4.21) 0.0156
COAD 263 0.41 (0.24, 0.68) <0.0006
DLBC 44 0.24 (0.08, 0.70) 0.0087
ESCA 154 1.66 (1.01, 2.73) 0.0453
GBM 131 9.05 (5.77, 14.18) <0.0001
HNSC 468 0.84 (0.54, 1.30) 0.4235
KICH 63 0.29 (0.13, 0.68) 0.0042
KIRC 503 0.86 (0.55, 1.36) 0.5328
KIRP 270 0.47 (0.27, 0.82) 0.0074
LGG 466 1.92 (1.21, 3.06) 0.0058
LIHC 333 1.48 (0.93, 2.37) 0.1016
LUAD 447 1.05 (0.67, 1.63) 0.8344
LUSC 413 0.68 (0.43, 1.06) 0.0909
MESO 64 3.39 (2.05, 5.60) <0.0001
OV 337 1.70 (1.11, 2.59) 0.0138
PAAD 166 2.88 (1.82, 4.56) <0.0001
PCPG 170 0.11 (0.04, 0.33) <0.0001
PRAD 490 0.05 (0.02, 0.12) <0.0001
READ 84 0.20 (0.08, 0.54) 0.0012
SARC 248 1.18 (0.75, 1.85) 0.4861
SKCM 97 1.15 (0.63, 2.08) 0.6531
STAD 314 1.39 (0.88, 2.19) 0.1525
TGCT 128 0.05 (0.01, 0.19) <0.0001
THCA 494 0.06 (0.03, 0.13) <0.0001
THYM 117 0.12 (0.04, 0.36) 0.0001
UCEC 162 0.54 (0.31, 0.96) 0.0369
UCS 53 2.50 (1.46, 4.29) 0.0009
UVM 74 1.18 (0.65, 2.14) 0.5788
DR score 8336 0.18 (0.11, 0.29) <0.0001
0.02 0.05 0.1 0.2 0.5 1 2 5 10
b
DR Group High Low
TCGA-KIRC
Log−rank P < 0.0001
Median follow-up time: 1268 (572, 1952) days
TCGA-LIHC
Log−rank P = 0.014
Median follow-up time: 632 (372, 1149) days
TCGA-LUAD
Log−rank P = 0.013
Median follow-up time: 669 (434, 1147) days
TCGA-GBM
Log−rank P = 0.040
Median follow-up time: 361 (167, 535) days
TCGA-COAD
Log−rank P = 0.0075
Median follow-up time: 682 (396, 1194) days
TCGA-HNSC
Log−rank P = 0.0052
Median follow-up time: 645 (384, 1172) days
TCGA-MESO
Log−rank P = 0.040
Median follow-up time: 536 (283, 860) days
Survival probability
Days
Number at risk
c
TCGA−LIHC
High−DR group vs Low−DR group
Total = 2209 DEGs
FZD1
G6PD
Up−regulated
Down−regulated
-log10 adjusted P−value
log2 fold change
