# Supplementary material for: An integrative pan-cancer analysis of the molecular characteristics of dietary restriction in tumour microenvironment
Source: eBioMedicine. 2024 Mar 19;102:105078. doi: 10.1016/j.ebiom.2024.105078 (PMC10965464; doi:10.1016/j.ebiom.2024.105078)
Supplement: Antibody validation file [file mmc3.docx]

Statement

In this study, commercial antibodies, including anti-FZD1 (rabbit polyclonal LS-A4150, 1:200, LS-A3484-50, LSBio), anti-G6PD (rabbit polyclonal, 1:500, ab993, Abcam) were used.

Detail documentation:

1. Anti-G6PD

RRID: AB_296714

Relevant reference:

Leung D, Price ZK, Lokman NA, Wang W, Goonetilleke L, Kadife E, Oehler MK, Ricciardelli C, Kannourakis G, Ahmed N. Platinum-resistance in epithelial ovarian cancer: an interplay of epithelial-mesenchymal transition interlinked with reprogrammed metabolism. J Transl Med. 2022;20(1):556.

Purchase website:

https://www.abcam.com/products/primary-antibodies/glucose-6-phosphate-dehydrogenase-antibody-ab993.html

Antibody registry: https://www.antibodyregistry.org/AB_296714

1. Anti-FZD1

RRID: AB_591407

Relevant reference:

Planutis K, Planutiene M, Nguyen AV, Moyer MP, Holcombe RF. Invasive colon cancer, but not non-invasive adenomas induce a gradient effect of Wnt pathway receptor frizzled 1 (Fz1) expression in the tumor microenvironment. J Transl Med. 2013;11:50.

Purchase website:

https://www.lsbio.com/pathplus-antibodies/pathplus-fzd1-antibody-frizzled-1-antibody-n-terminus-ihc-ls-a3484/1345

Antibody registry: https://www.antibodyregistry.org/AB_591407
